# A systems-based approach to analyse the host response in murine lung macrophages challenged with respiratory syncytial virus

**DOI:** 10.1186/1471-2164-14-190

**Published:** 2013-03-18

**Authors:** Laxmi Iyer Ravi, Liang Li, Richard Sutejo, Hui Chen, Pui San Wong, Boon Huan Tan, Richard J Sugrue

**Affiliations:** 1 Division of Molecular Genetics and Cell Biology, Nanyang Technological University, 60 Nanyang Drive, Singapore 637551, Singapore; 2 Singapore-MIT Alliance for Research & Technology (SMART), Centre for Life Sciences, 28 Medical Drive, Singapore 117456, Singapore; 3 Detection and Diagnostics Laboratory, DSO National Laboratories, 27 Medical Drive, Singapore, 117510, Singapore

**Keywords:** Respiratory syncytial virus, Macrophage transcriptome, Host response, Interferon, Cytokine induction

## Abstract

**Background:**

Respiratory syncytial virus (RSV) is an important cause of lower respiratory tract infection in young children. The degree of disease severity is determined by the host response to infection. Lung macrophages play an important early role in the host response to infection and we have used a systems-based approach to examine the host response in RSV-infected lung-derived macrophage cells.

**Results:**

Lung macrophage cells could be efficiently infected (>95%) with RSV *in vitro*, and the expression of several virus structural proteins could be detected. Although we failed to detect significant levels of virus particle production, virus antigen could be detected up until 96 hours post-infection (hpi). Microarray analysis indicated that 20,086 annotated genes were expressed in the macrophage cells, and RSV infection induced an 8.9% and 11.3% change in the global gene transcriptome at 4 hpi and 24 hpi respectively. Genes showing up-regulated expression were more numerous and exhibited higher changes in expression compared to genes showing down-regulated expression. Based on gene ontology, genes with cytokine, antiviral, cell death, and signal transduction functions showed the highest increases in expression, while signalling transduction, RNA binding and protein kinase genes showed the greatest reduction in expression levels. Analysis of the global gene expression profile using pathway enrichment analysis confirmed that up-regulated expression of pathways related to pathogen recognition, interferon signalling and antigen presentation occurred in the lung macrophage cells challenged with RSV.

**Conclusion:**

Our data provided a comprehensive analysis of RSV-induced gene expression changes in lung macrophages. Although virus gene expression was detected, our data was consistent with an abortive infection and this correlated with the activation of several antivirus signalling pathways such as interferon type I signalling and cell death signalling. RSV infection induced a relatively large increase in pro-inflammatory cytokine expression, however the maintenance of this pro-inflammatory response was not dependent on the production of infectious virus particles. The sustained pro-inflammatory response even in the absence of a productive infection suggests that drugs that control the pro-inflammatory response may be useful in the treatment of patients with severe RSV infection.

## Background

Human respiratory syncytial virus (RSV) is responsible for approximately 64 million infections and 160,000 deaths each year [1]. It is the most important cause of lower respiratory tract (LRT) infections in young children and neonates, and it is a significant cause of acute LRT-associated death in young children in developing countries [2]. In addition, several other high-risk groups include the elderly and immunocompromised adults. This overall clinical scenario is worsened by the lack of an available vaccine and the limited availability of specific therapeutic drugs.

The mature RSV particle comprises a ribonucleoprotein (RNP) complex that is surrounded by a protein shell formed by the matrix protein. The RNP complex consists of viral genomic RNA (vRNA), the nucleocapsid (N) protein, the phosphoprotein (P protein), M2-1 protein, and the large (L) protein [3-10]. The virus particle is surrounded by a lipid membrane in which the attachment (G) protein and fusion (F) proteins are inserted. During virus entry the G protein mediates attachment of the virus to the cell [11], while the F protein mediates the fusion of the virus and host cell membrane [12]. Two distinct virus structures are formed in RSV-infected epithelial cells that lead to a productive infection, called virus filaments and inclusion bodies. The virus filaments form at the plasma membrane on the surface of infected cells and are sites where the virus structural proteins interact to form mature filamentous virus particles. The inclusion bodies are the sites in the cell where the RNP-associated proteins and virus-specific RNA accumulate [13-15], suggesting that inclusion bodies may be accumulations of pre-assembled virus RNPs prior to packaging into the progeny virus.

LRT infection due to RSV is a complicated process and several cell types are implicated in the disease progression [16]. Disease severity due to extensive lung tissue damage correlates with enhanced pro-inflammatory cytokine secretion and inflammation. RSV infection of macrophage cells in the lungs of severely infected patients has been demonstrated [17], and these immune cells are proposed to play an important role in the early response to RSV infection [18-20]. The murine animal model system has been extensively used to examine the pathology of RSV infection since it is generally representative of the disease progression in the LRT of humans [17]. Several studies have employed blood monocyte derived macrophage (BMDM) cells as a model system to understand interactions between RSV and lung macrophages. However, the biological properties of macrophages is influenced by their tissue location [21], suggesting that BMDM cells and pulmonary macrophage cells may behave differently with respect to RSV infection. Since pulmonary macrophages play an important role during the initial stages of RSV infection we have performed a detailed molecular and cellular characterisation of RSV infection in primary murine lung macrophage cells. To obtain a better understanding of the interaction of RSV with lung-derived primary murine macrophages we also used a systems-based approach to examine the effect of RSV infection on the host transciptome during the early and late stages of infection.

## Results

### RSV exhibits similar replication characteristics in alveolar macrophage and pulmonary macrophage cells

**Figure 1:**
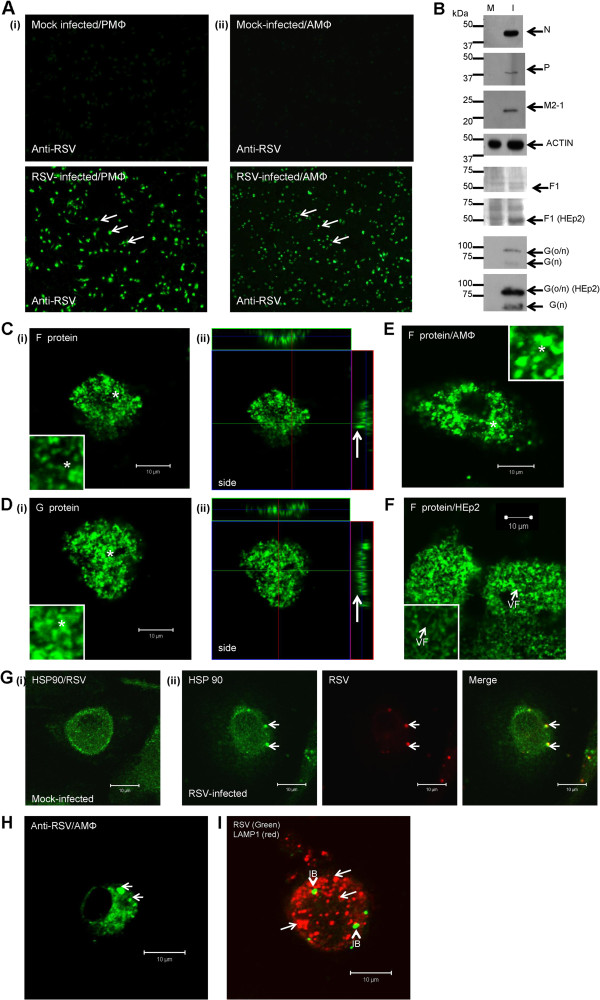
**Lung macrophages are efficiently infected with RSV. (A) (i) **PMΦ cells and **(ii) **AMΦ cells were mock-infected or RSV-infected, and at 24 hrs post-infection (hpi) stained with anti-RSV and examined using fluorescence microscopy (objective x 10) (highlighted by white arrows). **(B) **Mock (M) and RSV-infected (I) PMΦ cell lysates were examined by immunoblotting using anti-N, anti-P, anti-M2-1, anti-F or anti-G. Anti-actin is shown as the loading control. Analysis of mock and RSV-infected HEp2 cells (using same cell numbers) with anti-F and anti-G is shown. Protein bands of the expected sizes for the respective virus proteins are indicated. The 50 kDa F1 subunit, and the O- and N-linked (G(o/n)) and N-linked (G (n)) glycosylated G proteins are indicated. PMΦ cells were infected with RSV and at 24 hpi the cells were fixed, labelled using **(C) **anti-F or **(D) **anti-G and examined using florescence scanning confocal microscopy (FSCM) **(i) **at a focal plane that shows the cell surface staining (inset, highlighted by *) or **(ii) **in cross-section showing the surface staining (highlighted by white arrow). **(E) **The AMΦ cells were stained using anti-F and examined at a focal plane that shows the cell surface (inset, highlighted by *). **(F) **RSV-infected HEp2 cells at 24 hpi stained using anti-F (inset) and examined using FSCM, the virus filaments (VF) on the cell surface are highlighted. **(G) (i) **Mock and **(ii) **RSV-infected cells were stained with anti-RSV and anti-HSP90. The presence of virus-induced inclusion bodies and HSP90 staining of these structures is highlighted (white arrows). **(H**) RSV-infected AMΦ cells were stained using anti-RSV, and the presence of stained inclusion bodies highlighted (white arrow). **(I) **At 24 hpi RSV-infected PMΦ cells were stained with anti-RSV and anti-LAMP-1. The inclusion bodies (IB) and the LAMP-1 punctate staining pattern (white arrows) are highlighted.

Alveolar macrophage (AMΦ) cells represent a minor population of the total pulmonary lung macrophage population. However, although AMΦ cells may exhibit some distinct biological properties from the total lung macrophage population, they are expected to exhibit broadly similar tissue-specific biological properties to the bulk lung macrophage population [22,23]. We isolated an average of 2x10^4^ alveolar macrophage (AMФ) cells per mouse from broncho-alveolar lavage fluid, and 1x10^6^ pulmonary macrophage (PMФ) cells per mouse were isolated from whole lung tissue. These macrophage cell preparations were stained using antibodies which recognise murine macrophage antigenic markers CD11b, CD11c and F4/80 [24] and examined by fluorescence (IF) microscopy. Approximately 95% of the adhered cells showed CD11b and F4/80 staining, confirming their murine macrophage origin (Additional file 1: Figure S1A and B). The AMΦ and PMΦ cell preparations were infected with RSV and at 24 hpi, RSV-infected cells were stained with anti-RSV and imaged using IF microscopy. The numbers of stained cells (determined by fluorescence microscopy) and total cell numbers (determined by bright field microscopy) in the same field of view allowed an estimation of cell infection levels. The presence of virus antigen in >95% of the cells in either cell preparation indicated efficient infection (Figure 1A), suggesting that each cell preparation was similarly susceptible to RSV infection. The expression of virus proteins in the virus-infected PMФ cells was further confirmed by immunoblotting infected cell lysates using anti-N, anti-P anti-G, anti-F(MAb169) and anti-M2-1 which revealed protein species of the expected sizes (Figure 1B).

Virus-infected PMΦ cells were labelled either with anti-F or anti-G and examined using fluorescence scanning confocal microscopy (FSCM) (Figure 1C and D), which revealed a structured punctuate staining pattern on the surface of infected cells in each case. A similar staining pattern was also observed on anti-F labelled RSV-infected AMФ cells (Figure 1E). In permissive cell types such as HEp2 cells the infectious particles form filamentous structures that allow cell to cell transmission. However, in both cases this surface staining pattern observed in macrophage cells was distinct from the anti-F stained filamentous virus particles that form on the permissive HEp2 cell line (Figure 1F). Scanning electron microscopy (SEM) revealed the presence of membrane ruffling on both mock-and virus-infected PMΦ cells, with increased membrane ruffling following virus infection (Additional file 2: Figure S2A). Similarly, transmission electron microscopy (TEM) revealed numerous membrane protrusions on mock-infected and RSV-infected cells (Additional file 2: Figure S2B), with increased appearance of these structures on virus-infected cells. Both electron microscopy techniques indicated increased membrane ruffling during virus infection but the absence of progeny virus.

Examination of anti-RSV stained PMΦ cells revealed cytoplasmic structures that were similar in appearance to inclusion bodies (Figure 1G). The presence of heat shock protein 90 (HSP90) within inclusion bodies has been reported [25], and co-staining of infected PMΦ cells with anti-RSV and anti-HSP90 indicated the presence of HSP90 in these structures (Figure 1G (ii)). A similar staining pattern was also detected within RSV-infected AMФ cells labelled with anti-RSV (Figure 1H). The PMΦ cells exhibited phagocytic activity (Additional file 1: Figure S1C), and the presence of LAMP1 in mature phagosomes has been established [26]. To distinguish the inclusion bodies from phagosomes the infected cells were co-stained with anti-LAMP1 and anti-RSV, and examined using FSCM (Figure 1I). Co-staining with anti-LAMP1 was not observed in these structures suggesting that the anti-RSV staining pattern was due to inclusion bodies and not phagocytosed virus antigen.

Similar vRNA levels at between 2.5 and 20 hpi were detected (Additional file 3: Figure S3A) suggesting low levels of vRNA synthesis. The virus infectivity recovered in RSV-infected HEp2 and PMΦ cells was compared by plaque assay which indicated virus titres of 3x10^6^ pfu/ml and 2x10^1^ pfu/ml respectively. This low level of virus recovered from the infected PMΦ cells was likely due to residual virus from the input virus inoculum. Similarly the tissue culture supernatant (TCS) of RSV-infected HEp2 and PMΦ cells was added to HEp2 cells and anti-RSV stained cells detected using IF microscopy. An abundance of stained cells was observed with the TCS of the infected HEp2 cells indicating efficient progeny virus production. In contrast using this assay we failed to observe stained cells using the TCS of RSV-infected AMΦ cells and only sporadic stained cells were detected using the TCS of RSV-infected PMΦ cells (Additional file 3: Figure S3B).

Collectively although these data indicated that RSV infection results in the formation of virus antigen and the production of inclusion bodies, efficient infectious virus particle production does not occur. This is consistent with previous observations indicating abortive infection in lung macrophages [27]. These data also suggest that the AMΦ and PMΦ cells preparations behave similarly with respect to challenge with RSV.

### Global gene expression changes in RSV-infected pulmonary macrophage (PMΦ) cells

Macrophage cell activation is associated with changes in the macrophage transcriptome leading to cell reprogramming [28,29]. The effect of RSV infection on the host cell transcriptome was examined using the GeneChip^®^ Mouse Genome 430 2.0 Array (Affymetrix) high density microarray system. This provides a sensitive method to monitor virus-induced changes in the global gene expression profile at the early stages in infection. The AMΦ and PMΦ cell preparations behaved similarly with regards to RSV infection, however due to logistical difficulties in obtaining sufficient AMФ cells for the microarray analysis, the gene expression analysis was restricted to the PMФ cell preparation.

**Figure 2:**
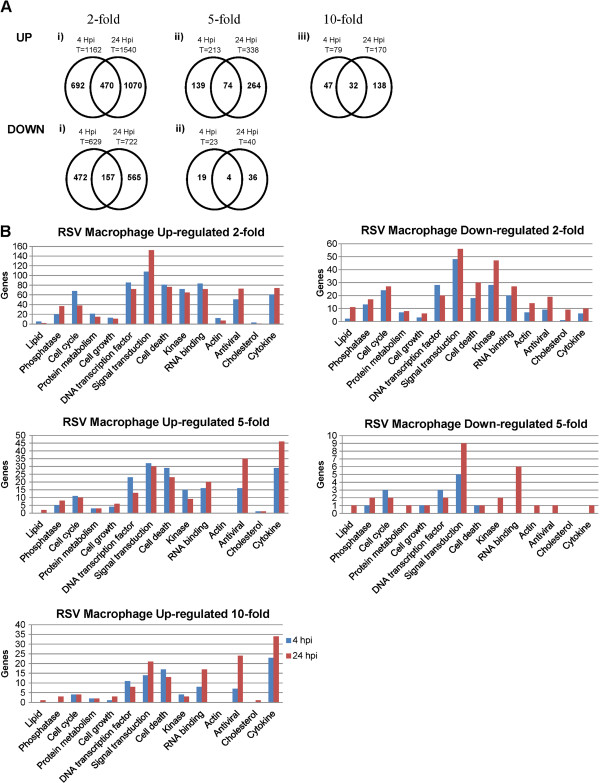
**Temporal global host cell expression changes during RSV-infection. (A) **The genes showing up-regulated and down-regulated gene expression that are unique to either 4 or 24 hrs post-infection (hpi) or common to both 4 and 24 hpi are indicated in the venn diagram. T is the total number of genes at each time using the fold change cut-off. **(B) **The numbers of genes based on gene ontology showing up-regulated or down-regulated expression in the microarray analysis of RSV-infected MΦ cells are summarized. The genes showing changes in expression levels were filtered based on ≥2-fold, ≥5-fold and ≥10-fold changes (P ≤ 0.05). Genes showing ≥10 fold down-regulated expression following virus infection were not detected.

There are 28,974 annotated genes on the microarray system, and analysis of the microarray data indicated that 69.3% of the annotated genes were expressed in mock-infected cells. This indicated that a relatively large proportion of the total transcriptome was represented in our analysis. The effect of RSV infection on host gene expression microarray at early (4 hpi) and late (24 hpi) stages in the virus infection was analysed (Figure 2A). An 8.9% change in the global host gene transcriptome was observed at 4 hpi, with 1162 and 629 host genes showing up-regulated and down-regulated gene expression respectively. At 24 hpi this increased to 11.3% of the total host cell transcriptome, with 1540 and 722 host genes showing up-regulated and down-regulated gene expression respectively. Collectively these data indicated that RSV infection caused a relatively minor change in the global host-gene expression profile by 24 hpi.

The genes showing virus-induced changes in expression were filtered using the fold-change (FC) in expression levels, which allowed us to distinguish the genes showing a high FC in expression levels (>10 FC). A significant proportion of genes showing changes in expression levels following RSV infection were uniquely expressed at either time point. The genes showing up-regulated expression and down-regulated expression following virus infection were compared at both times of infection using a 2-FC cut-off. Common up-regulated genes and down-regulated genes accounted for 17.4% and 12.8% total gene changes respectively. This suggested that distinct changes in host cell transcriptome occurred at early and late stages in infection. In general genes showing up-regulated expression exhibited a higher fold-change (FC) in expression levels compared to those genes showing down-regulated expression, and most differentially expressed genes showed a higher FC at 24 hpi compared with that at 4 hpi. Based on gene ontology the largest proportion of genes showing a greater than 10-FC increase in expression levels were functionally defined as cytokine, antiviral, signal transduction and cell-death related, while those showing the greatest reduction in expression were signal transduction and RNA binding (Figure 2B).

**Figure 3:**
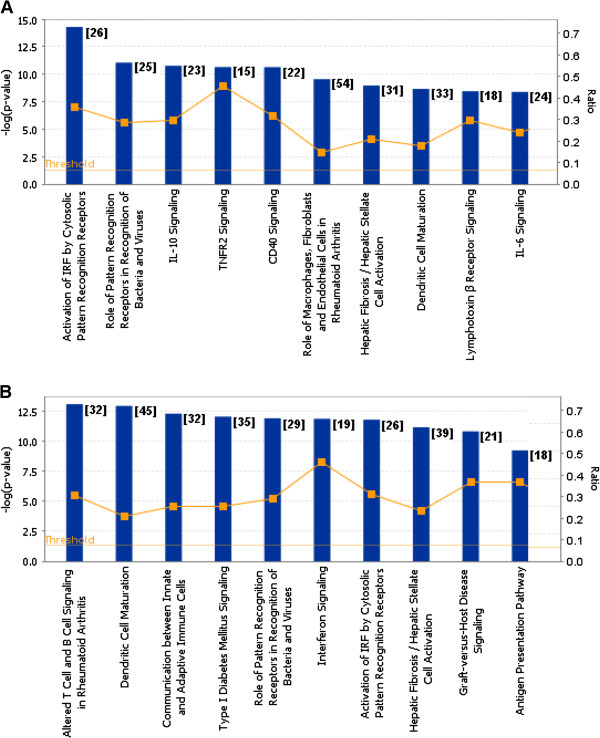
**Different canonical pathways in RSV-infected PMФ cells based on macrophage host genes showing differential gene expression (A) 4 and (B) 24 hours post-infection (hpi). **The microarray data sets for each time of infection was processed using GeneSpring GX 11.0 and uploaded into Ingenuity Pathways Analysis version 2012 (IPA). The data was filtered based on the significance cutoff ≤0.05 and fold change cutoff ≥2. Genes were categorized using IPA, and p-values were calculated by Fisher’s exact test for each canonical pathway at 4 and 24 hpi, and the top 10 statistically significant canonical pathways were identified at each time of infection and listed. The number of genes listed for each pathway is indicated in the parentheses. The orange boxes indicate the ratio (Ratio) of the numbers of genes that show changes in gene expression data and the total number of genes in the respective canonical pathway. Threshold was set at p-value = 0.05 and indicated as –log (p-value) on the Y-axis and the X-axis shows the terms of each canonical pathway.

Gene enrichment analysis based on the global gene expression changes was performed using the ingenuity pathway analysis (IPA). This was performed at each time of infection, and the canonical pathways identified at each time point were ranked based on the corresponding p value (statistical significance) using a cut-off of p < 0.05. This enabled us to obtain a list of the 10 most significant pathway groupings at 4 hpi (Figure 3A) and 24 hpi (Figure 3B). A comparison of the numbers of genes showing changes in gene expression with the total number of genes in these canonical pathways indicated ratios of between approximately 0.15 and 0.5 (i.e. between 15 and 50% of the total number of genes in these pathways). This indicated that a relatively large number of genes were represented in these canonical pathways. Although in general most pathways were represented at each time of infection (e.g. pathways involving genes that activate pathogen pattern recognition receptors), their ranking in the list varied (Additional file 4: Table S1). Collectively, this analysis indicates that RSV infection induces a range of antivirus responses in macrophages, from those playing a role in initial host responses involving pathogen recognition, cytokine signalling (e.g. interferon induction) and antigen presentation.

Our analysis indicated that genes and pathways that were up-regulated were more highly represented in the global gene analysis, both in terms of gene numbers and FC in expression levels. To examine pathways that showed down-regulated expression a pathway enrichment analysis was performed on all genes that were grouped separately into either up-regulated or down-regulated expression at either time point. We generated a pathway list showing the 15 most significant pathways in each category (Additional file 5: Table S2). In the list, antivirus response pathways were again more highly represented at both 4 and 24 hpi for pathways showing up-regulated gene expression. In contrast, a similar pathway enrichment analysis on genes showing down-regulated expression indicated genes in pathways defined as involved in rho signalling and virus entry via endocytosis were more highly represented at 4 hpi. At 24 hpi pathways involved in cholesterol biosynthesis and lipid metabolism were more highly represented, indicating that by 24 hpi virus infection may also induce changes in pathways playing a role in cellular metabolism.

### Validation of selected gene expression changes in RSV-infected pulmonary macrophage (PMΦ) cells

Since genes that play a role in the antivirus responses showed the highest FC in gene expression we examined the gene expression changes of specific antivirus networks in more detail. These virus-induced changes in gene expression were also validated using independent biochemical assays.

### Cytokine gene expression

**Figure 4:**
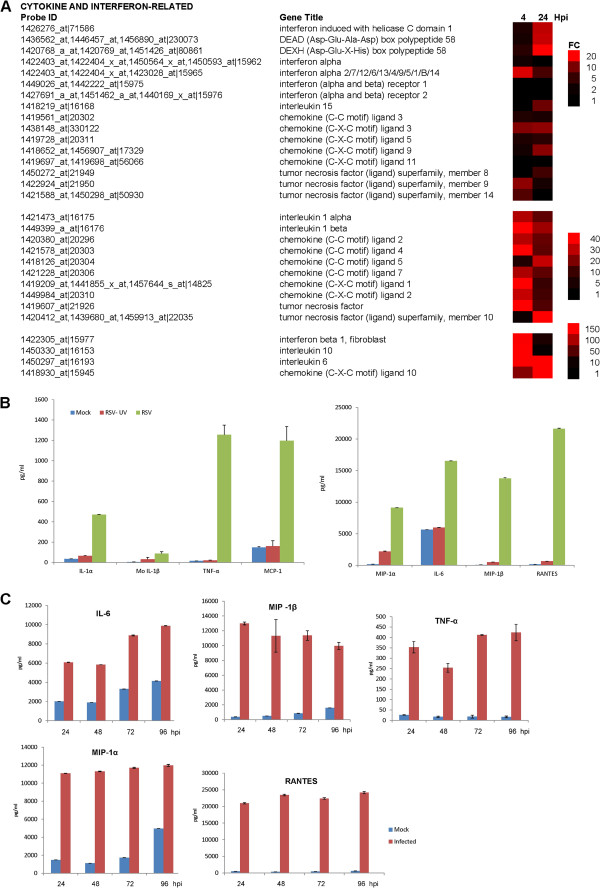
**Changes in cytokine gene expression profiles in RSV-infected macrophage cells. (A)** The fold change (FC) in cytokine gene expression at 4 and 24 hours post-infection (hpi) is presented as a heat map. In each case the FC range, probe identification (Probe ID), and gene name (Gene Title) are indicated. **(B) **Increased cytokine secretion in RSV-infected PMΦ cells. The PMФ cell preparation was either mock-infected or infected using a RSV inoculum that was either non-treated (RSV) or UV-inactivated (RSV UV). At 24 hours post-infection the cytokine levels were measured in the tissue culture supernatant. **(C) **The levels of specific cytokines persisted in RSV-infected PMΦ cells. Cells were infected with RSV and the cytokine levels measured at between 24 and 96 hpi. In each case the tissue culture supernatant was harvested and the cytokine levels measured using the Bio-Plex Mouse Cytokine 23-plex panel. The data shown were obtained from triplicate measurements, and representative data from one of two independent experiments are shown.

RSV infection was associated with increased expression of several cytokine genes (Figure 4A). Increased expression of some specific cytokine genes (e.g. CCL4, IL-10, TNF- α) could be detected at 4 hpi, which is consistent with previous reports suggesting early detection of these cytokines in RSV-infected cells [30-35]. However, in general the levels of these early expressed cytokines declined by 24 hpi. In contrast, other cytokines only showed increased expression at 24 hpi (e.g. CCL5), indicating late or delayed expression.

The protein levels of some specific cytokines were measured in the tissue culture supernatant TCS of mock-infected and RSV-infected MΦ cells. Increased levels of MIP-1α (CCL3), MIP-1β (CCL4), MCP-1(CCL2), IL1β, IL1α, RANTES (CCL5), TNFα and IL-6 were observed by 24 hpi (Figure 4B). This was consistent with the increased cytokine gene expression levels detected in the microarray analysis. The cytokine levels in PMΦ cells challenged with infectious virus (RSV-NT) and ultra-violet inactivated virus (RSV-UVT) were compared to confirm that cytokine secretion was dependant on the presence of infectious virus. The HEp2 cell line is highly susceptible to RSV infection and we failed to observe significant anti-RSV labelling of HEp2 cells that were challenged with RSV-UVT confirming virus inactivation (Additional file 6: Figure S4). Similar cytokine levels in the TCS of mock-infected and RSV-UVT-infected cells were observed, and a comparison of the cytokine levels induced in RSV-NT-infected and RSV-UVT-infected PMΦ cells indicated 60% lower levels in IL-6, and between a 100-fold and 1000-fold lower levels of several other cytokines (e.g. RANTES and TNFα) in cells challenged with RSV-UVT (Figure 4B). A similar analysis on the RSV-infected AMΦ cells showed increased MIP-1α, MIP-1β, RANTES levels and smaller increases in IL6 and TNFα secretion when challenged with infectious RSV. Mock-infected and RSV-UVT-infected cells showed similar cytokine levels in the TCS, indicating that cytokine induction only occurred in AMΦ cells challenged with infectious virus (Additional file 7: Figure S5). Although the levels of cytokines measured in the AMΦ cell preparation were lower than that measured in the PMФ cell preparation, these data indicated the increased secretion of several common pro-inflammatory cytokines in both cell preparations that have been implicated in RSV-mediated lung pathology in humans [34]. These data also indicated that that infectious virus was required to induce these changes in cytokine induction and cytokine signalling.

RSV-infected PMΦ cells were further examined to determine if the increased cytokine levels could be detected at later stages in the infection process. We observed elevated levels of these cytokine in the TCS of RSV-infected cells up to 96 hpi (Figure 4C). We used 96 hpi as the cut-off in this analysis since after this time we noted significant deterioration in the appearance and condition of the macrophage preparation. However, this analysis indicated that the virus-induced increases in cytokine levels were sustained even in the absence of significant levels of progeny virus production. Although at 24 hpi some of the cytokine gene expression levels were lower than that measured at 4 hpi, a sustained increase in cytokine gene expression at 24 hpi was still apparent, which may partly account for the sustained cytokine levels measured in the TCS.

### Interferon type I signalling and interferon stimulated gene expression (ISG)

Interferon proteins are important cytokine mediators of the innate immune response, whose expression is induced following recognition of specific pathogen-associated molecular patterns (PAMPs) by pattern recognition receptors (PRRs). These PRRs include DExD/H 58, also known as retinoic acid inducible gene I (RIG-I), and interferon-induced helicase C domain-containing protein 1, also known as melanoma differentiation-associated gene-5 (mda-5). A small increase in RIG-I and mda-5 gene expression was detected at 4 hpi, but significantly larger increases in RIG-I and mda-5 gene expression was observed by 24 hpi. The increased RIG-I and mda-5 gene expression correlated with a small initial increase in IFNα gene expression (Figure 4A), but a significantly higher increase in IFNβ gene expression. At 4 hpi a 150-FC in IFNβ gene expression was recorded, which declined to a 17-FC increase by 24 hpi. The microarray data was supported by qPCR measurements of the IFNβ mRNA in mock and virus-infected cells (Additional file 8: Figure S6 (i)), which indicated an approximate 220-FC and 30-FC in expression at 4 and 24 hpi respectively. Both methods showed the same magnitude of gene expression changes, and similar trends in gene expression changes. Although there was a variation in FC values obtained using microarray analysis and qPCR analysis, both methods showed consistent trends in IFNβ gene expression. The differences observed in FC values in gene expression levels obtained using these two methods has been the subject of several reports [36,37].

**Figure 5:**
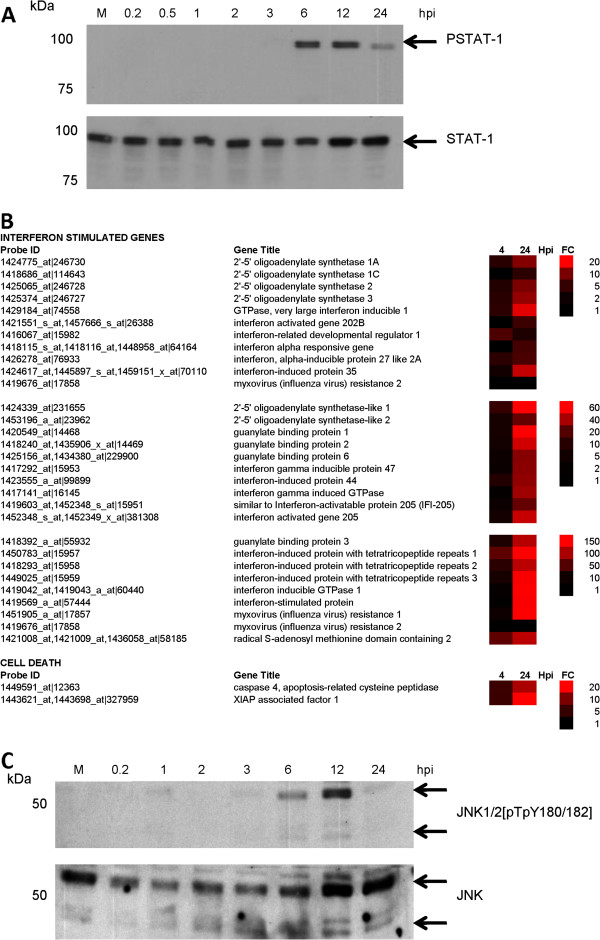
**Changes in interferon stimulated gene (ISG) expression and cell death signalling in RSV-infected macrophage cells. (A) **Activation of the STAT-1 signalling pathway in RSV-infected PMΦ cells. Cells were infected with RSV and at between 0.2 and 24 hrs post-infection hpi the cells were harvested and the relative STAT-1 and PSTAT-1 levels determined by immunoblotting using relevant antibodies. **(B) **The fold change (FC) in the expression of interferon stimulated genes and cell death signalling related genes at 4 and 24 hpi in virus-infected cells compared with mock-infected cells is represented as a heat map. In each case the FC range, the probe identification (Probe ID) and gene name (Gene Title) are indicated. **(C)** Activation of the JNK signalling pathway in RSV-infected PMΦ cells. Cells were infected with RSV and at between 0.2 hpi and 24 hpi the cells were harvested and the relative JNK and phosphorylated JNK levels determined by immunoblotting using relevant antibodies. The mock-infected cell lysate (M) was harvested at 12 hpi.

Increased Type I IFN (IFN α/β) expression would be expected to lead to STAT-1 activation, and the activation status of the STAT-1 in RSV-infected PMΦ cells was examined. RSV-infected cells were harvested at between 0.2 and 24 hpi and the presence of STAT-1 and phosphorylated STAT-1 (pSTAT-1) was detected at each time-point by immunoblotting with appropriate antibodies (Figure 5A). Comparable levels of STAT-1 were detected at all the time points examined, while pSTAT-1 was detected at between 3 and 6 hpi. Although the STAT-1 levels did not decline during the course of infection, a reduction in pSTAT-1 levels was observed from between 12 and 24 hpi. The reason for this reduction in pSTAT-1 is currently uncertain, but was consistent with a decline in IFNβ mRNA levels indicated by both the qPCR and microarray data.

Activation of the STAT-1 would be expected to lead to the expression of several IFN stimulated genes (ISG) (reviewed in [38]). Although the microarray analysis indicated basal levels of ISG expression at 4 hpi, relatively large increases in ISG expression levels was detected at 24 hpi (Figure 5B), which was consistent with STAT-1 activation. The increased ISGs detected included several ISGs with relatively well characterized anti-virus activities (e.g. Myxovirus (MX) protein and RSAD2), and several other ISGs with less well characterized anti-virus activities (e.g. IFN-induced protein 44). The microarray analysis was validated using qPCR to compare the mRNA levels of several selected ISGs in mock and infected cells (Additional file 8: Figure S6 (i)).

### Cell death signalling

The increased expression of XIAP associated factor 1 (XAF1) was detected in the microarray analysis (Figure 5B), and XAF1 is a pro-apoptotic protein that inhibits the anti-apoptotic activities of the XIAP [39,40]. JNK activation can be mediated via Type I IFN signalling pathways [41], and the role of ISG products have been implicated in p38 and JNK activation [42,43]. Furthermore, XAF1 expression is enhanced by activated JNK [44], and the activation status of the JNK pathway was therefore examined in RSV-infected MФ cells. Cells were infected with RSV and at between 12 mins post-infection and 24 hpi the presence of JNK and phosphorylated JNK (pJNK) was detected (Figure 5C). Significant pJNK levels was only detected at 6 hpi, the pJNK levels appeared to decline by 24 hpi in a similar manner which may be related to the decline in IFNβ mRNA levels at 24 hpi. A role for JNK activation in regulating apoptosis has been proposed (reviewed in [45]), and the correlation between JNK activation and enhanced expression of XAF1 is consistent with JNK playing a role in the induction of cell-death pathways in RSV-infected PMФ cells.

### Macrophage activation and antigen presentation gene expression

**Figure 6:**
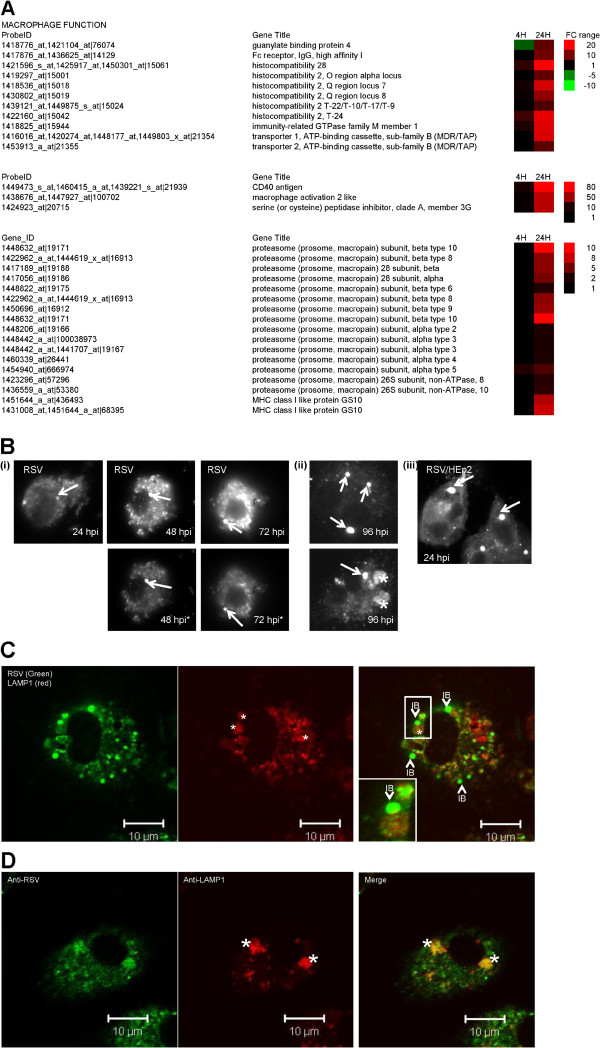
**Increased expression of genes involved in macrophage activation and antigen presentation occurs in RSV-infected macrophage cells. (A) **The fold change (FC) in the expression of cytokine at 4 and 24 hours post-infection (hpi) are compared with that in mock-infected cells as a heat map. In each case, the FC range, probe identification (Probe ID), and gene name (Gene Title) are indicated. **(B)** RSV-infected PMΦ cells were stained with anti-RSV and examined by fluorescence microscopy at **(i) **24 hpi, 48 hpi and 72 hpi and **(ii) **96 hpi. The stained cells were examined using either the same camera exposure time or at a reduced exposure time (highlighted by *) to enable the inclusion bodies (IB) to be viewed (at magnification x20). Inclusion bodies are highlighted (white arrow) and a more diffuse anti-RSV staining pattern is highlighted (*). **(iii) **RSV-infected HEp2 cells stained using anti-RSV at 24 hpi. The IBs are highlighted (white arrow). **(C) **At 48 hpi RSV-infected PMΦ cells were stained with anti-RSV and anti-LAMP-1. The inclusion bodies (IB) and the LAMP-1 punctate staining pattern (*) are highlighted **(D)**. Also shown is the co-staining pattern of anti-RSV and anti-LAMP-1 in the diffusely stained phagosomes (*) that predominates latter in infection.

Several genes involved in macrophage activation and antigen presentation showed up-regulated gene expression at 24 hpi (Figure 6A). These genes included CD40 antigen, macrophage activation 2 like gene expression, and transporter 1 ATP-binding cassette (TAP1). The up-regulated expression of several genes encoding proteosomal proteins that play an important role in inflammation antigen presentation [46,47] was also detected at 24 hpi. The microarray analysis was validated using qPCR, which showed an approximate 35-fold increase in the CD40 and TAP1 gene expression by 24 hpi (Additional file 8: Figure S6(ii)), consistent with the gene enrichment analysis indicating the presence of antigen-presentation pathways at 24 hpi.

Examination of infected cells by IF microscopy revealed that inclusion bodies could be detected in virus-infected PMФ cells up to 96 hpi (Figure 6B (i) and (ii),), indicating that these virus structures persisted in infected macrophage cells at later times in the infection process, and even in the absence of infectious virus production. The inclusion bodies detected at 24 hpi were considerably smaller than those observed in RSV-infected HEp2 cells at a similar time of infection (Figure 6B (iii)). This indicated a slower rate of inclusion body formation in PMФ cells, consistent with slow rates of vRNA gene transcription in these cells. We noted that from 24 hpi an additional diffuse anti-RSV staining pattern was observed which became more prominent at 96 hpi (Figure 6B (ii), highlighted by *). This was distinct from the more compact staining pattern observed for inclusion bodies and we presumed that this alternative staining pattern could arise due to the phagocytosis of virus antigen. Infected cells were co-stained with anti-RSV and anti-LAMP1 and while the inclusion bodies did not co-localise with anti-LAMP1 (Figure 6C), we noted extensive co-localisation of anti-LAMP1 with these diffusely anti-RSV staining structures (Figure 6D). This suggested that this alternative staining pattern indicated sites within the cell where engulfment of virus antigen by mature phagosomes occurs, and was consistent with the up-regulated expression of several genes involved in proteosomal degradation and antigen presentation.

## Discussion

Disease severity due to RSV infection is likely to be partly determined by a combination of the genetic characteristics of the host [48] and the capacity of different RSV strains to induce pro-inflammatory cytokines[49]. In addition RSV can cause a persistent infection in susceptible hosts correlating with prolonged disease symptoms [50]. Histopathology of fatal cases of RSV infection have demonstrated infection of lung macrophage cells [17], although no evidence for the presence of infectious RSV particles was noted. Our observations are consistent with abortive replication in lung macrophages, and is consistent with *in vitro* studies on mouse [27] and human lung macrophage cells [51-53]. However, in contrast to these previous studies our study demonstrated virus gene expression and the formation of inclusion bodies, the latter being a characteristic of productive RSV infection. However, it appears that cellular processes are activated during virus infection that block the formation of infectious virus particles. Therefore, although all the available data suggests that lung macrophages are an important source of pro-inflammatory cytokines during RSV infection, they may not significantly contribute to virus propagation in the lower airway.

The capacity of RSV to overcome the IFN antivirus response and replicate in epithelial cells has been described [54,55]. The interaction between STAT-1 and STAT-2 is required for IFN signalling and these previous studies suggest inhibition of type 1 interferon occurs by down-regulation of STAT2 expression. The virus-induced STAT signalling that we observe in RSV infected macrophages suggests that down regulated expression of the STAT proteins did not occur in lung macrophage cells. The precise mechanism that leads to the inability of RSV to counter the IFN response in pulmonary macrophage cells is currently unclear and will require further examination. However, our data suggests that infectious virus particles are required for inducing the host response to infection, suggesting that initial events related to an early stage in the RSV replication cycle initiates the antivirus responses. The correlation between IFN signalling and abortive infection suggests that this response may play a role in restricting the formation of infectious virus. The expression of several ISGs with proven anti-viral activities was recorded [56,57], but it is currently unclear if one or more these ISGs block the formation of mature RSV particles that leads to the abortive infection in lung macrophages.

In addition to ISG expression we noted the up-regulated expression of several genes involved in proteosomal degradation and antigen presentation. It is expected that this process leads to the display of RSV-related peptides on the surface of the macrophage cells (i.e. antigen presentation). Although we were able to detect strong fluorescence staining of the virus surface glycoproteins on surface of infected macrophages, we failed to detect the presence of similar levels of the corresponding proteins by western blotting. This suggests that the virus glycoproteins may undergo proteolytic degradation, and since the expression of the virus glycoproteins is required for generating infectious virus this may also partly account for the abortive infection in these cells. In contrast, the presence of numerous virus-induced inclusion bodies was detected later in the virus infection, suggesting that the polymerase associated proteins persisted in infected cells. This was consistent with the ready detection of the RNP-associated N, P and M2-1 proteins by immunoblotting of cell lysates prepared from infected cell lysates. The available data suggests that inclusion bodies may represent sites of virus genome transcription (and virus gene replication), and that sequestering these proteins into the RNPs may protect these proteins from proteosomal degradation during the initial phase of infection (up to 24 hpi). Although the inclusion bodies are largely engulfed within phagosomes during the later stages of infection, the formation of these structures early in infection may provide increased resistance of the virus RNP-associated virus proteins to proteosomal degradation. Thus the presence of the inclusion bodies even in the presence of a potent antivirus response may play a role in the persistence of immunopathologic symptoms that have been reported in RSV-infected mice [50].

Although recent evidence suggests that circulating RSV strains exhibit slow rates of evolution [58], they may also differ in their capacity to induce pro-inflammatory cytokines [49]. Our analysis of the infected lung macrophage cells indicates a sustained pro-inflammatory cytokine response in the absence of productive infection. This suggests that strategies that can control the pro-inflammatory response may be useful in the treatment of patients with LRT RSV infection. Future studies will use the *in vitro* macrophage cell system described here to characterise the host response of different clinical strains. Careful analysis of transcriptome responses in field isolates should improve our understanding of the interaction between RSV and macrophages during the initial stages of LRT infection. This may lead to the identification of important cell signalling pathways that mediate the host anti-virus response to infection, which in turn could lead to the identification of novel drug targets to control the pro-inflammatory responses during RSV infection.

## Conclusions

This is the first study that has used global gene expression to examine the host response in RSV-infected murine lung macrophages. Our data shows that although RSV challenge leads to an abortive infection, virus antigen within inclusion bodies are formed. RSV challenge leads to up-regulated expression of genes involved in pathogen recognition, interferon signalling, inflammation and macrophage activation. The sustained pro-inflammatory cytokine levels even in the absence of a productive infection may help explain the persistence of symptoms that are associated with LRTI. In addition our data suggests that drugs that control the pro-inflammatory response may be useful in the treatment of patients with severe RSV infection.

## Methods

### Virus and tissue culture

The RSV A2 strain was used throughout this study and was prepared using HEp2 cell culture [25]. RSV particles were recovered from tissue culture media by centrifugation at 150,000g for 2 hr at 4°C, after which the virus was gently and uniformly resuspended in an equal volume of fresh DMEM with 2% FCS at 4°C. The infectivity of the resulting inoculum was confirmed using a HEp2 cell microplaque assay [59,60]. Unless specified the cells were infected using a multiplicity of infection of 4. UV-inactivated virus was prepared at 4°C by exposing the virus inoculum (at a distance of 1cm) to a UV radiation source (λ = 256 nm) for 30 mins. Murine lung CD11b + cells were prepared from 6–8 weeks old special pathogen-free (SPF) female Balb/c mice as described previously [61] but with modifications. The lungs were digested with collagenase D (1 mg/ml; Gibco) and single cell suspension (0.5% BSA, 2mM EDTA, in 1XPBS) obtained was passed through a 30 μm filter. CD11b + cells were purified using CD11b microbeads and a LS positive selection column (Miltenyi Biotec). Broncho-alveolar lavage was performed to harvest aveolar macrophages (AMФ) cells from the mouse lungs. The lungs were washed five times with 1ml PBS each time and the cells were isolated by centrifugation at 300g for 10mins. Cell viability assays were performed using Trypan blue which confirmed greater than 95% cell viability. The cells were cultured in L929 cell conditioned (30%v/v) medium for 3 days at 37°C in 5% CO_2_. Prior to use, the cells were washed using PBS to remove non-adherent cells which further enhanced the purification of these cell preparations.

### Ethical approval

This study was carried out in strict accordance with the recommendations in the Guidelines on the Care and Use of Animals for Scientific Purposes of the National Advisory Committee for Laboratory Animal Research (NACLAR), Singapore. The protocol was approved by the Institutional Animal Care and Use Committee, National University of Singapore (Approved Protocol Number: 046/09). All the operations on animals were done after the euthanasia of the animals by CO_2_ inhalation, and all efforts were made to minimize animal suffering.

### Antibodies and specific reagents

The use of the F protein (MAb 169), M2-1 protein, N protein and P protein antibodies have been described previously [62]. The anti-F was obtained from Geraldine Taylor (IAH, UK), anti-G and anti-LAMP-1 were purchased from Abcam, and anti-RSV (RCL3) from Novacastra Laboratories. The HSP90 antibody was purchased from Santa Cruz Laboratories. The CD11b-FITC and F4/80-FITC antibodies were purchased from Miltenyi Biotec and Biolegend respectively.

### Immunofluorescence microscopy

Cells on 13 mm glass coverslips were fixed with 3% paraformaldehyde (PFA) in PBS for 30 min at 4°C, permeabilised using 0.1% saponin, and labelled using virus-specific primary antibodies and the appropriate secondary antibodies. The mouse and rabbit secondary antibodies were purchased from Molecular Probes as described previously [62]. The stained cells were mounted on slides using Citifluor and visualized using either a Nikon eclipse 80i fluorescence microscope, or a Zeiss Axioplan 2 confocal microscope using appropriate machine settings. Cells stained with CD11b-FITC and F4/80-FITC antibodies were labelled prior to PFA fixation.

### Western blotting

Cell lysates were prepared in 1X boiling mix (1% SDS, 15% glycerol, 1% β-mercaptoethanol, 60mM sodium phosphate, pH 6 · 8) at 100°C for 2 min. The protein samples were transferred by western blotting onto PVDF membrane, which were blocked with PBS containing 5%(w/v) skimed milk powder (Marvel™) as described previously (McDonald et al. 2004). The membrane was incubated with the appropriate primary antibody and anti-mouse IgG (whole molecule) peroxidase conjugate (Sigma, USA). The protein bands were visualized using the ECL protein detection system (Amersham, USA), and the apparent molecular masses were estimated using Kaleidoscope protein standards (Bio Rad, USA).

### Cytokine assay

Supernatant from the macrophage cells were centrifuged at 10,000g for 10min at 4°C after which the supernatant was used for cytokine assay. Cytokines present in the media were analysed with the Bio-Plex Protein Array System (BioRad) using the Bio-Plex Mouse Cytokine 23-Plex Panel (1 x 96-well, # M60009RDPD, Bio-Rad) according to the manufacturer’s instructions.

### Real-time quantitative PCR

Total RNA was extracted from cells at 4°C using the RNeasy kit (Qiagen, USA) and reverse-transcribed using Superscript II (Invitrogen, USA). Primers for cell-specific genes were designed using the Probefinder software (http://qpcr.probefinder.com/organism.jsp) from the Universal Probe Library (UPL) Design Center (Roche). The comprehensive list of primer and probe sequences are detailed (Additional file 9: Table S3). Quantitative real-time PCR (qPCR) was carried out with the iCycler System (BioRad) following the protocol previously described [63]. The sequences of the elongation factor EEF1A1 (*H. sapiens*) was used as the reference gene since. Both absolute and relative quantification analysis were done using comparative Ct (ΔΔCt method) [64]. The vRNA copy number was determined as follows. Briefly, both N gene representing the vRNA copy number and EF gene were PCR-amplified, gel-extracted and quantified. Serial dilutions of each were made and measured to determine the Ct values. All copy numbers presented in the result are calculated based on 10^4^ EF copy number. Relative FC of the host virus gene expression were calculated with respect to the mock-infected cells and normalized with the corresponding cell line’s EF gene.

### Microarray analysis

The mouse genome-wide gene expression was examined using the GeneChip^®^ Mouse Genome 430 2.0 Array (Affymetrix) high density microarray systems. The cells were harvested at 4°C using RNAlater (Ambion) in PBS buffer. Total RNA was extracted using the RNeasy minikit (Qiagen). Double-stranded cDNA was synthesized from 3 μg of total RNA with the GeneChip One-Cycle cDNA synthesis kit (Invitrogen, Affymetrix), followed by synthesis of biotin-labelled cRNA using the GeneChip IVT labelling kit (Affymetrix). After fragmentation, 15 μg of labelled cRNA was hybridized to the arrays, which were washed and stained using the GeneChip Fluidics Station 450 (Affymetrix), and then scanned with the GeneChip scanner 3000 (Affymetrix). Quality control, GeneChip hybridization and data acquisition were performed according to the standard protocols available from Affymetrix. Normalization using a global scaling strategy to a target intensity of 500 was first performed using GCOS (v1.1, Affymetrix) before uploading the .CHP data file into GeneSpring GX 11.0 (Agilent) for data analysis. Further normalizations were carried out in GeneSpring: (a) per chip normalization to the 50th percentile and (b) per gene normalization where RSV-infected samples were normalized to mock-infected samples. Genes were selected for statistical analysis according to the following criteria: (i) only genes that were flagged as present in all three replicates (mock- or RSV-infected), and (ii) a FC of ≥2 or ≤ -2 between RSV- and mock-infected samples in all triplicate microarray experiments. Finally, a one-way analysis of variance (ANOVA) of the above selected genes was performed with a P-value cut-off of less than 0.05 to determine significantly up- and down-regulated genes during RSV infection. The function, biological processes and pathways of these genes were then examined using the GeneSpring program. All microarray data was deposited as MIAME-compliant data submissions GSE31378 in the Gene Expression Omnibus. Functional interpretation of differentially expressed genes was analyzed and these genes were grouped based on their biological function and cellular component as annotated by Gene Ontology (GO). The known pathways of differentially expressed genes associated with metabolism and signalling were investigated by canonical pathway analysis using Ingenuity Pathways Analysis (IPA; Ingenuity Systems http://www.ingenuity.com). Those differentially expressed genes with known gene IDs and corresponding expression fold changes were uploaded into the software. P-value was used to determine the probability that the association between the genes in the dataset and the canonical pathway. IPA uses a right-tailed Fisher’s exact test to calculate p-value for canonical pathway analysis, with a p-value cut-off of ≤ 0.05.

### Electron microscopy

a) Scanning electron microscopy. Cells grown on glass coverslips were critically point dried (Polaron CPD) prior to mounting on aluminium stubs and carbon-coated using an Edwards sputter coater device [60]. The cells were visualized with a Jeol 5600 using appropriate machine settings. b) Transmission Electron Microscopy. Cells were processed as described previously [60]. Briefly, cell monolayers were pelleted in BEEM capsules (TAAB) and fixed with 2.5% (v/v) glutaraldehyde in PBS at 4°C. The cell pellet was post-fixed with 1% (w/v) osmium tetroxide solution (TAAB) and dehydrated through a gradient of ethanol concentrations. The cell pellet was infiltrated with epon 812 (TAAB Laboratories), and heat polymerized at 65°C for 24 hr. Ultrathin sections were stained using uranyl acetate (saturated in 50:50 ethanol/water), counter-stained with lead citrate and examined in a Jeol 1400 transmission electron microscope.

## Competing interests

The authors have declared that no competing interests exist.

## Authors’ contributions

Conceived and designed the experiments: RJS. Performed the experiments: LL, LIR, RS, PSW, CR, BHT. Analysed data: CH RS. Wrote the paper: RJS. Assisted in manuscript preparation BHT. All authors read and approved the final manuscript.

## Supplementary Material

Additional file 1: Figure S1 Antigenic characterization of lung macrophage cells. (**A**) PMΦ cells were labelled using anti-CD11b and anti-F4/80 and (**B**) AMΦ cells were labelled with anti-CD11b and anti-CD11c and examined using immunofluorescence (IF) microscopy or bright field microscopy (BF) (at magnification x10). (**C**) The PMΦ cells exhibited phagocytic activity. Polystyrene latex beads (2.0-μm diameter) (Sigma) were coated with BSA (Sigma Aldrich) as described previously (May et al., 2000). Briefly, the beads were washed three times in PBS, incubated with BSA (10mg/ml) at 4°C overnight with gentle rotation, and then washed to remove excess BSA at room temperature for 1 hour with rotation, and then washed again with PBS. The PMΦ cells were plated on cover slips placed and the latex beads were added at approximately 20 particles/cell for 30 minutes at 33^0^C. Non-internalized beads were removed by washing with PBS, the cells fixed using 3% PFA in PBS and visualized using an inverted fluorescent microscope (Nikon) (objective x100). Click here for file

Additional file 2: Figure S2 Ultrastructural analysis of RSV-infected PMΦ cells by electron microscopy. Mock and RSV-infected MΦ cells at 24 hour post-infection (hpi) were processed for (**A**) scanning electron microscopy (SEM) or (**B**) transmission electron microscopy (TEM). Representative images are shown. Membrane ruffling and protrusions on the surface of the MΦ cells imaged by SEM and TEM respectively are highlighted (black arrows). SEM, magnification at x5,000;TEM, magnification at x40,000. Click here for file

Additional file 3: Figure S3 Infectious virus particles are not produced in RSV-infected macrophages (**A**) The total RNA was extracted from RSV-infected MΦ cells at 2.5 and 24 hpi and the vRNA levels estimated by qPCR as described in methods. This is the average of 3 measurements and p < 0.05. (**B**) The tissue culture supernatant (TCS) from mock-infected or RSV-infected HEp2 cells or PMΦ cells was harvested at 24 hpi and used to infect HEp2 monolayers. At 24 hpi the presence of infected cells in the HEp2 cell monolyer was stained using anti-RSV and viewed by fluorescence microscopy (anti-RSV) and bright field microscopy (BF) (objective x10). Click here for file

Additional file 4: Table S1 Pathway enrichment analysis based on global macrophage host genes showing changes in gene expression following RSV infection at 4 and 24 hpi. Macrophages were infected with RSV at two different time points and IPA version 2012 software was applied for pathway analysis. The 10 most significant canonical pathways enriched by global gene expression (p*-*value ≤ 0.05 and FC ≥ 2) at 4 hpi and 24 hpi are listed. Corresponding p-values, gene numbers and individual genes are also indicated. Click here for file

Additional file 5: Table S2 Pathway enrichment analysis based on macrophage host genes showing up-regulated and down-regulated gene expression following infection with RSV at 4 and 24 hpi. Macrophages were infected with RSV at two different time points and IPA version 2012 software was applied for pathway analysis. Significant canonical pathways enriched by differentially up-regulated or down-regulated genes (p*-*value ≤ 0.05 and FC ≥ 2) at 4 hpi and 24 hpi were listed. Corresponding p-values, gene numbers and individual genes were also represented. The pathway in bold indicated that it is commonly enriched at both 4 hpi and 24 hpi. Click here for file

Additional file 6: Figure S4 UV-treatment inactivates RSV infectivity. The RSV inoculum was either non-treated (RSV-NT) or UV-treated (RSV-UVT) and used to infect either HEp2 cells and at 24 hours post-infection the cells were stained using anti-RSV and viewed using a Nikon eclipse 80i fluorescence microscope (objective x10). Click here for file

Additional file 7: Figure S5 Cytokine induction in the murine alveolar macrophage (AMФ) cell preparation. The AMФ cell preparation was either mock-infected (Mock) or infected using a RSV inoculum that was either non-treated (RSV) or UV-inactivated (RSV(UV)). The levels of pro-inflammatory cytokines in the tissue culture supernatant (TCS) was measured at 24 hrs post-infection (hpi). In each case the data shown were obtained from triplicate measurements (p < 0.05) and representative data from two independent experiments is shown. Click here for file

Additional file 8: Figure S6 Validation in the differential gene expression of selected genes in RSV-infected MΦ cells using qPCR. The relative mRNA levels in mock and RSV-infected MΦ cells of (**i**) 2’,5’-oligoadenylate synthase 2 (OAS2), radical S-adenosyl methionine domain containing 2 (RSAD2), RANTES, Interferon-β (IFNβ) and 2’,5’-oligoadenylate synthase-like genes (OASL) (insets show expression levels in mock-infected cells) and (**ii**) TAP1, CD40. The average values obtained from three independent measurements (p < 0.05) and representative data from one experiment. Click here for file

Additional file 9: Table S3 Primer and probes sequences designed for real-time qPCR. Primer sequences and UPL probes (Roche) used for real-time qPCR validation . RSV N gene, IFN-β1: interferon β; OAS2: 2’, 5’-oligoadenylate synthase 2; OASL: 2’, 5’-oligoadenylate synthase-like; RSAD2: radical S-adenosyl methionine domain containing 2; HMGCR: 3-hydroxy-3-methyl-glutaryl-CoA reductase; CH25H: cholesterol 25-hydroxylase; TAP1: Antigen peptide transporter 1; CD40:CD40; RANTES (CCL5); EF: elongation factor. Click here for file

## References

[B1] WHO WHO Initiative for Vaccine Research. Acute Respiratory Infections (Update September 2009) Available at http://www.who.int/vaccine_research/diseases/ari/en/index2.html Accessed 13 August 2010

[B2] Nair H Nokes DJ Gessner BD Dherani M Madhi SA Singleton RJ O’Brien KL Roca A Wright PF Bruce N Global burden of acute lower respiratory infections due to respiratory syncytial virus in young children: a systematic review and meta-analysis Lancet 2010 375 9725 1545 1555 10.1016/S0140-6736(10)60206-1 20399493 PMC2864404

[B3] Collins PL Hill MG Cristina J Grosfeld H Transcription elongation factor of respiratory syncytial virus, a nonsegmented negative-strand RNA virus Proc Natl Acad Sci USA 1996 93 1 81 85 10.1073/pnas.93.1.81 8552680 PMC40182

[B4] Grosfeld H Hill M Collins P RNA replication by respiratory syncytial virus (RSV) is directed by the N, P, and L proteins; transcription also occurs under these conditions but requires RSV superinfection for efficient synthesis of full-length mRNA J Virol 1995 69 9 5677 5686 7637014 10.1128/jvi.69.9.5677-5686.1995 PMC189426

[B5] Yu Q Hardy RW Wertz GW Functional cDNA clones of the human respiratory syncytial (RS) virus N, P, and L proteins support replication of RS virus genomic RNA analogs and define minimal trans-acting requirements for RNA replication J Virol 1995 69 4 2412 2419 7884888 10.1128/jvi.69.4.2412-2419.1995 PMC188915

[B6] Jin H Cheng X Zhou HZY Li S Seddiqui A Respiratory Syncytial Virus That Lacks Open Reading Frame 2 of the M2 Gene (M2-2) Has Altered Growth Characteristics and Is Attenuated in Rodents J Virol 2000 74 1 74 82 10.1128/JVI.74.1.74-82.2000 10590093 PMC111515

[B7] Bermingham A Collins PL The M2–2 protein of human respiratory syncytial virus is a regulatory factor involved in the balance between RNA replication and transcription Proc Natl Acad Sci USA 1999 96 20 11259 11264 10.1073/pnas.96.20.11259 10500164 PMC18021

[B8] Fearns R Collins PL Role of the M2-1 Transcription Antitermination Protein of Respiratory Syncytial Virus in Sequential Transcription J Virol 1999 73 7 5852 5864 10364337 10.1128/jvi.73.7.5852-5864.1999 PMC112646

[B9] Ghildyal R Mills J Murray M Vardaxis N Meanger J Respiratory syncytial virus matrix protein associates with nucleocapsids in infected cells J Gen Virol 2002 83 Pt 4 753 757 11907323 10.1099/0022-1317-83-4-753

[B10] Hardy RW Wertz GW The product of the respiratory syncytial virus M2 gene ORF1 enhances readthrough of intergenic junctions during viral transcription J Virol 1998 72 1 520 526 9420254 10.1128/jvi.72.1.520-526.1998 PMC109403

[B11] Levine S Klaiber-Franco R Paradiso PR Demonstration that glycoprotein G is the attachment protein of respiratory syncytial virus J Gen Virol 1987 68 Pt 9 2521 2524 3655746 10.1099/0022-1317-68-9-2521

[B12] Scheid A Choppin PW Two disulfide-linked polypeptide chains constitute the active F protein of paramyxoviruses Virology 1977 80 1 54 66 10.1016/0042-6822(77)90380-4 195398

[B13] Garcia J Garcia-Barreno B Vivo A Melero JA Cytoplasmic inclusions of respiratory syncytial virus-infected cells: formation of inclusion bodies in transfected cells that coexpress the nucleoprotein, the phosphoprotein, and the 22K protein Virology 1993 195 1 243 247 10.1006/viro.1993.1366 8317099

[B14] Santangelo PJ Bao G Dynamics of filamentous viral RNPs prior to egress Nucleic Acids Res 2007 35 11 3602 3611 10.1093/nar/gkm246 17485480 PMC1920244

[B15] Carromeu C Simabuco FM Tamura RE Farinha Arcieri LE Ventura AM Intracellular localization of human respiratory syncytial virus L protein Arch Virol 2007 152 12 2259 2263 10.1007/s00705-007-1048-4 17703289

[B16] Collins PL Graham BS Viral and host factors in human respiratory syncytial virus pathogenesis J Virol 2008 82 5 2040 2055 10.1128/JVI.01625-07 17928346 PMC2258918

[B17] Johnson JE Gonzales RA Olson SJ Wright PF Graham BS The histopathology of fatal untreated human respiratory syncytial virus infection Mod Path 2007 20 1 108 119 10.1038/modpathol.3800725 17143259

[B18] Haeberle HA Takizawa R Casola A Brasier AR Dieterich HJ Van Rooijen N Gatalica Z Garofalo RP Respiratory syncytial virus-induced activation of nuclear factor-kappaB in the lung involves alveolar macrophages and toll-like receptor 4-dependent pathways J Infect Dis 2002 186 9 1199 1206 10.1086/344644 12402188

[B19] Reed JL Brewah YA Delaney T Welliver T Burwell T Benjamin E Kuta E Kozhich A McKinney L Suzich J Macrophage Impairment Underlies Airway Occlusion in Primary Respiratory Syncytial Virus Bronchiolitis J Infect Dis 2008 198 12 1783 1793 10.1086/593173 18980502

[B20] Pribul PK Harker J Wang B Wang H Tregoning JS Schwarze J Openshaw PJ Alveolar macrophages are a major determinant of early responses to viral lung infection but do not influence subsequent disease development J Virol 2008 82 9 4441 4448 10.1128/JVI.02541-07 18287232 PMC2293049

[B21] Gersuk GM Razai LW Marr KA Methods of in vitro macrophage maturation confer variable inflammatory responses in association with altered expression of cell surface dectin-1 J Immunol Methods 2008 329 1–2 157 166 17997408 10.1016/j.jim.2007.10.003

[B22] Takeuchi O Akira S Innate immunity to virus infection Immunol Rev 2009 227 1 75 86 10.1111/j.1600-065X.2008.00737.x 19120477 PMC5489343

[B23] Lohmann-Matthes M Steinmuller C Franke-Ullmann G Pulmonary macrophages Eur Respir J 1994 7 9 1678 1689 7995399

[B24] Khazen W M’Bika JP Tomkiewicz C Benelli C Chany C Achour A Forest C Expression of macrophage-selective markers in human and rodent adipocytes FEBS Lett 2005 579 25 5631 5634 10.1016/j.febslet.2005.09.032 16213494

[B25] Radhakrishnan A Yeo D Brown G Myaing MZ Iyer LR Fleck R Tan BH Aitken J Sanmun D Tang K Protein analysis of purified respiratory syncytial virus particles reveals an important role for heat shock protein 90 in virus particle assembly Mol Cell Proteomics 2010 9 1829 1848 10.1074/mcp.M110.001651 20530633 PMC2938102

[B26] Flannagan RS Jaumouille V Grinstein S The cell biology of phagocytosis Annu Rev Pathol 2012 7 61 98 10.1146/annurev-pathol-011811-132445 21910624

[B27] Franke-Ullmann G Pfortner C Walter P Steinmuller C Lohmann-Matthes M Kobzik L Freihorst J Alteration of pulmonary macrophage function by respiratory syncytial virus infection in vitro J Immunol 1995 154 1 268 280 7995946

[B28] Ricciardi-Castagnoli P Granucci F Opinion: Interpretation of the complexity of innate immune responses by functional genomics Nat Rev Immunol 2002 2 11 881 889 10.1038/nri936 12415311

[B29] Mosser DM Edwards JP Exploring the full spectrum of macrophage activation Nat Rev Immunol 2008 8 12 958 969 10.1038/nri2448 19029990 PMC2724991

[B30] Panuska JR Merolla R Rebert NA Hoffmann SP Tsivitse P Cirino NM Silverman RH Rankin JA **Respiratory syncytial virus induces interleukin-10 by human alveolar macrophages.** Suppression of early cytokine production and implications for incomplete immunity J Clin Invest 1995 96 5 2445 2453 10.1172/JCI118302 7593633 PMC185897

[B31] Garofalo R Sabry M Jamaluddin M Yu R Casola A Ogra P Brasier A Transcriptional activation of the interleukin-8 gene by respiratory syncytial virus infection in alveolar epithelial cells: nuclear translocation of the RelA transcription factor as a mechanism producing airway mucosal inflammation J Virol 1996 70 12 8773 8781 8971006 10.1128/jvi.70.12.8773-8781.1996 PMC190974

[B32] Zhang Y Luxon BA Casola A Garofalo RP Jamaluddin M Brasier AR Expression of respiratory syncytial virus-induced chemokine gene networks in lower airway epithelial cells revealed by cDNA microarrays J Virol 2001 75 19 9044 9058 10.1128/JVI.75.19.9044-9058.2001 11533168 PMC114473

[B33] Haeberle HA Kuziel WA Dieterich H-J Casola A Gatalica Z Garofalo RP Inducible Expression of Inflammatory Chemokines in Respiratory Syncytial Virus-Infected Mice: Role of MIP-1{alpha} in Lung Pathology J Virol 2001 75 2 878 890 10.1128/JVI.75.2.878-890.2001 11134301 PMC113984

[B34] McNamara PS Flanagan BF Hart CA Smyth RL Production of Chemokines in the Lungs of Infants with Severe Respiratory Syncytial Virus Bronchiolitis J Infect Dis 2005 191 8 1225 1232 10.1086/428855 15776367

[B35] Guerrero-Plata A Casola A Garofalo RP Human Metapneumovirus Induces a Profile of Lung Cytokines Distinct from That of Respiratory Syncytial Virus J Virol 2005 79 23 14992 14997 10.1128/JVI.79.23.14992-14997.2005 16282501 PMC1287587

[B36] Dallas PB Gottardo NG Firth MJ Beesley AH Hoffmann K Terry PA Freitas JR Boag JM Cummings AJ Kees UR Gene expression levels assessed by oligonucleotide microarray analysis and quantitative real-time RT-PCR – how well do they correlate? BMC Genomics 2005 6 59 10.1186/1471-2164-6-59 15854232 PMC1142514

[B37] Yuen T Wurmbach E Pfeffer RL Ebersole BJ Sealfon SC Accuracy and calibration of commercial oligonucleotide and custom cDNA microarrays Nucleic Acids Res 2002 30 10 e48 10.1093/nar/30.10.e48 12000853 PMC115302

[B38] Randall RE Goodbourn S Interferons and viruses: an interplay between induction, signalling, antiviral responses and virus countermeasures J Gen Virol 2008 89 1 1 47 10.1099/vir.0.83391-0 18089727

[B39] Liston P Fong WG Kelly NL Toji S Miyazaki T Conte D Tamai K Craig CG McBurney MW Korneluk RG Identification of XAF1 as an antagonist of XIAP anti-Caspase activity Nat Cell Biol 2001 3 2 128 133 10.1038/35055027 11175744

[B40] Holcik M Gibson H Korneluk R XIAP: Apoptotic brake and promising therapeutic target Apoptosis 2001 6 4 253 261 10.1023/A:1011379307472 11445667

[B41] Zhou Z Hamming OJ Ank N Paludan SR Nielsen AL Hartmann R Type III interferon (IFN) induces a type I IFN-like response in a restricted subset of cells through signaling pathways involving both the Jak-STAT pathway and the mitogen-activated protein kinases J Virol 2007 81 14 7749 7758 10.1128/JVI.02438-06 17507495 PMC1933366

[B42] Iordanov MS Paranjape JM Zhou A Wong J Williams BRG Meurs EF Silverman RH Magun BE Activation of p38 Mitogen-Activated Protein Kinase and c-Jun NH2-Terminal Kinase by Double-Stranded RNA and Encephalomyocarditis Virus: Involvement of RNase L, Protein Kinase R, and Alternative Pathways Mol Cell Biol 2000 20 2 617 627 10.1128/MCB.20.2.617-627.2000 10611240 PMC85147

[B43] Li G Xiang Y Sabapathy K Silverman RH An apoptotic signaling pathway in the interferon antiviral response mediated by RNase L and c-Jun NH2-terminal kinase J Biol Chem 2004 279 2 1123 1131 14570908 10.1074/jbc.M305893200

[B44] Wang J Zhang W Zhang Y Chen Y Zou B Jiang B Pang R Gu Q Qiao L Lan H c-Jun N-terminal kinase (JNK1) upregulates XIAP-associated factor 1 (XAF1) through interferon regulatory factor 1 (IRF-1) in gastrointestinal cancer Carcinogenesis 2009 30 2 222 229 19056926 10.1093/carcin/bgn271

[B45] Liu J Lin A Role of JNK activation in apoptosis: A double-edged sword Cell Res 2005 15 1 36 42 10.1038/sj.cr.7290262 15686625

[B46] Kloetzel PM Ossendorp F Proteasome and peptidase function in MHC-class-I-mediated antigen presentation Curr Opin Immunol 2004 16 1 76 81 10.1016/j.coi.2003.11.004 14734113

[B47] Qureshi N Vogel SN Papasian CJ Qureshi AA Morrison DC Van Way C, 3rd The proteasome: a central regulator of inflammation and macrophage function Immunol Res 2005 31 3 243 260 10.1385/IR:31:3:243 15888915

[B48] Janssen R Bont L Siezen CL Hodemaekers HM Ermers MJ Doornbos G Wijmenga C Goeman JJ Kimpen JL van ’t Slot R Genetic susceptibility to respiratory syncytial virus bronchiolitis is predominantly associated with innate immune genes J Infect Dis 2007 196 6 826 834 10.1086/520886 17703412

[B49] Levitz R Wattier R Phillips P Solomon A Lawler J Lazar I Weibel C Kahn JS Induction of IL-6 and CCL5 (RANTES) in human respiratory epithelial (A549) cells by clinical isolates of respiratory syncytial virus is strain specific Virol J 2012 9 190 10.1186/1743-422X-9-190 22962966 PMC3463437

[B50] Schwarze J O’Donnell DR Rohwedder A Openshaw PJM Latency and Persistence of Respiratory Syncytial Virus Despite T Cell Immunity Am J Respir Crit Care Med 2004 169 7 801 805 10.1164/rccm.200308-1203OC 14742302

[B51] Becker S Quay J Soukup J Cytokine (tumor necrosis factor, IL-6, and IL-8) production by respiratory syncytial virus-infected human alveolar macrophages J Immunol 1991 147 12 4307 4312 1753101

[B52] Becker S Soukup J Yankaskas JR Respiratory syncytial virus infection of human primary nasal and bronchial epithelial cell cultures and bronchoalveolar macrophages Am J Respir Cell Mol Biol 1992 6 4 369 374 10.1165/ajrcmb/6.4.369 1550681

[B53] Cirino NM Panuska JR Villani A Taraf H Rebert NA Merolla R Tsivitse P Gilbert IA Restricted replication of respiratory syncytial virus in human alveolar macrophages J Gen Virol 1993 74 Pt 8 1527 1537 8345347 10.1099/0022-1317-74-8-1527

[B54] Lo MS Brazas RM Holtzman MJ Respiratory Syncytial Virus Nonstructural Proteins NS1 and NS2 Mediate Inhibition of Stat2 Expression and Alpha/Beta Interferon Responsiveness J Virol 2005 79 14 9315 9319 10.1128/JVI.79.14.9315-9319.2005 15994826 PMC1168759

[B55] Ramaswamy M Shi L Varga SM Barik S Behlke MA Look DC Respiratory syncytial virus nonstructural protein 2 specifically inhibits type I interferon signal transduction Virology 2006 344 2 328 339 10.1016/j.virol.2005.09.009 16216295

[B56] Kochs G Haller O Interferon-induced human MxA GTPase blocks nuclear import of Thogoto virus nucleocapsids Proc Natl Acad Sci 1999 96 5 2082 2086 10.1073/pnas.96.5.2082 10051598 PMC26740

[B57] Kochs G Haller O GTP-bound Human MxA Protein Interacts with the Nucleocapsids of Thogoto Virus (Orthomyxoviridae) J Biol Chem 1999 274 7 4370 4376 10.1074/jbc.274.7.4370 9933640

[B58] Kumaria R Iyer LR Hibberd ML Simoes EA Sugrue RJ Whole genome characterization of non-tissue culture adapted HRSV strains in severely infected children Virol J 2011 8 372 10.1186/1743-422X-8-372 21794174 PMC3166936

[B59] Cannon MJ Microplaque immunoperoxidase detection of infectious respiratory syncytial virus in the lungs of infected mice J Virol Methods 1987 16 4 293 301 10.1016/0166-0934(87)90014-0 3312262

[B60] Sugrue RJ Brown C Brown G Aitken J McL. Rixon HW Furin cleavage of the respiratory syncytial virus fusion protein is not a requirement for its transport to the surface of virus-infected cells J Gen Virol 2001 82 6 1375 1386 11369882 10.1099/0022-1317-82-6-1375

[B61] Perrone LA Plowden JK Garcia-Sastre A Katz JM Tumpey TM H5N1 and 1918 pandemic influenza virus infection results in early and excessive infiltration of macrophages and neutrophils in the lungs of mice PLoS Pathog 2008 4 8 e1000115 10.1371/journal.ppat.1000115 18670648 PMC2483250

[B62] McDonald TP Pitt AR Brown G Rixon HWM Sugrue RJ Evidence that the respiratory syncytial virus polymerase complex associates with lipid rafts in virus-infected cells: a proteomic analysis Virology 2004 330 1 147 157 10.1016/j.virol.2004.09.034 15527841

[B63] Spackman E Senne DA Myers TJ Bulaga LL Garber LP Perdue ML Lohman K Daum LT Suarez DL Development of a Real-Time Reverse Transcriptase PCR Assay for Type A Influenza Virus and the Avian H5 and H7 Hemagglutinin Subtypes J Clin Microbiol 2002 40 9 3256 3260 10.1128/JCM.40.9.3256-3260.2002 12202562 PMC130722

[B64] Schmittgen TD Livak KJ Analyzing real-time PCR data by the comparative CT method Nat Protocols 2008 3 6 1101 1108 10.1038/nprot.2008.73 18546601

