# Supplementary figures and images for: A systems-based approach to analyse the host response in murine lung macrophages challenged with respiratory syncytial virus
Source: BMC Genomics. 2013 Mar 18;14:190. doi: 10.1186/1471-2164-14-190 (PMC3618260; doi:10.1186/1471-2164-14-190)

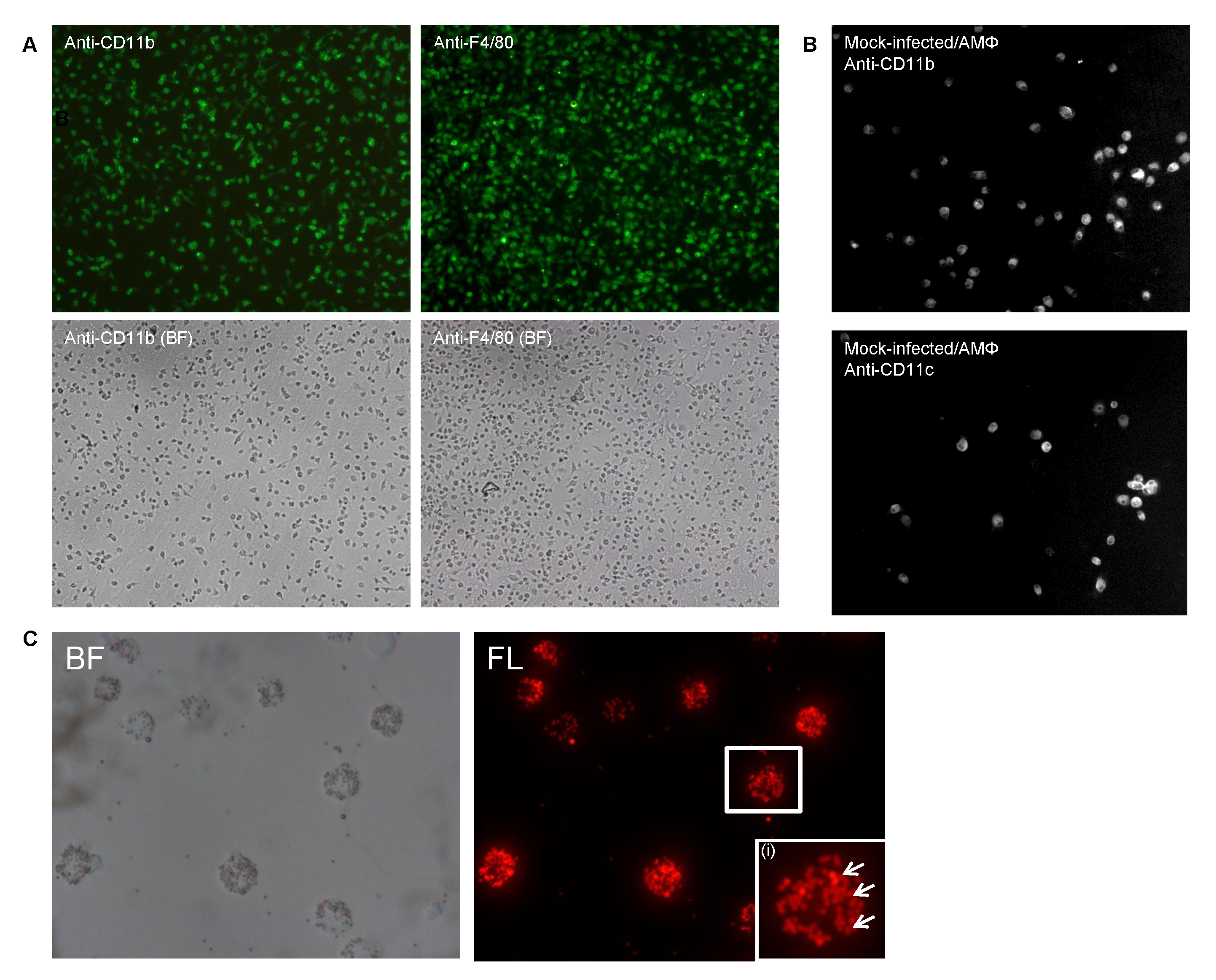

Supplement: Additional file 1: Figure S1 — Antigenic characterization of lung macrophage cells. (A) PMΦ cells were labelled using anti-CD11b and anti-F4/80 and (B) AMΦ cells were labelled with anti-CD11b and anti-CD11c and examined using immunofluorescence (IF) microscopy or bright field microscopy (BF) (at magnification x10). (C) The PMΦ cells exhibited phagocytic activity. Polystyrene latex beads (2.0-μm diameter) (Sigma) were coated with BSA (Sigma Aldrich) as described previously (May et al., 2000). Briefly, the beads were washed three times in PBS, incubated with BSA (10mg/ml) at 4°C overnight with gentle rotation, and then washed to remove excess BSA at room temperature for 1 hour with rotation, and then washed again with PBS. The PMΦ cells were plated on cover slips placed and the latex beads were added at approximately 20 particles/cell for 30 minutes at 330C. Non-internalized beads were removed by washing with PBS, the cells fixed using 3% PFA in PBS and visualized using an inverted fluorescent microscope (Nikon) (objective x100). [file 1471-2164-14-190-S1.tiff]

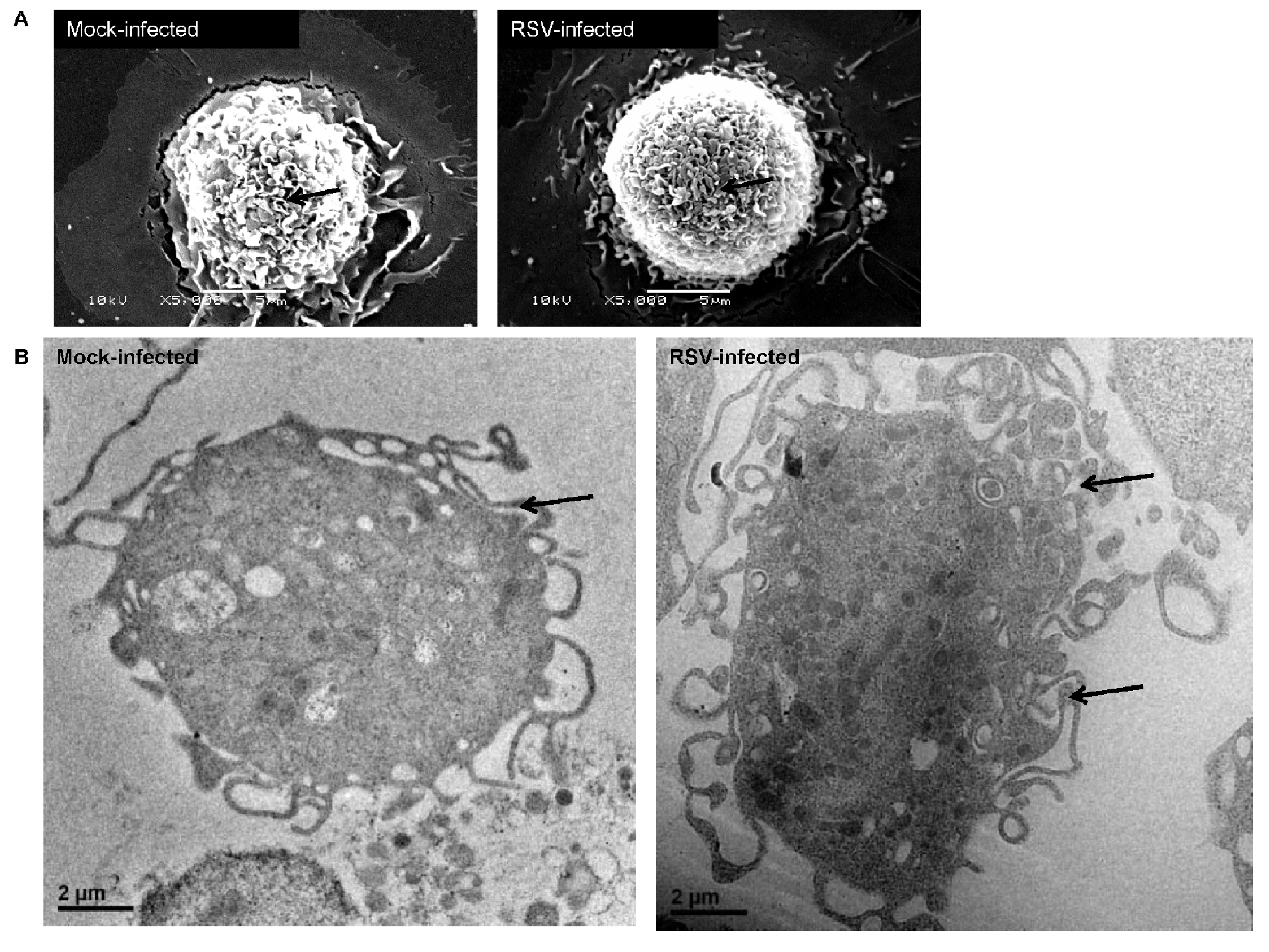

Supplement: Additional file 2: Figure S2 — Ultrastructural analysis of RSV-infected PMΦ cells by electron microscopy. Mock and RSV-infected MΦ cells at 24 hour post-infection (hpi) were processed for (A) scanning electron microscopy (SEM) or (B) transmission electron microscopy (TEM). Representative images are shown. Membrane ruffling and protrusions on the surface of the MΦ cells imaged by SEM and TEM respectively are highlighted (black arrows). SEM, magnification at x5,000;TEM, magnification at x40,000. [file 1471-2164-14-190-S2.tiff]

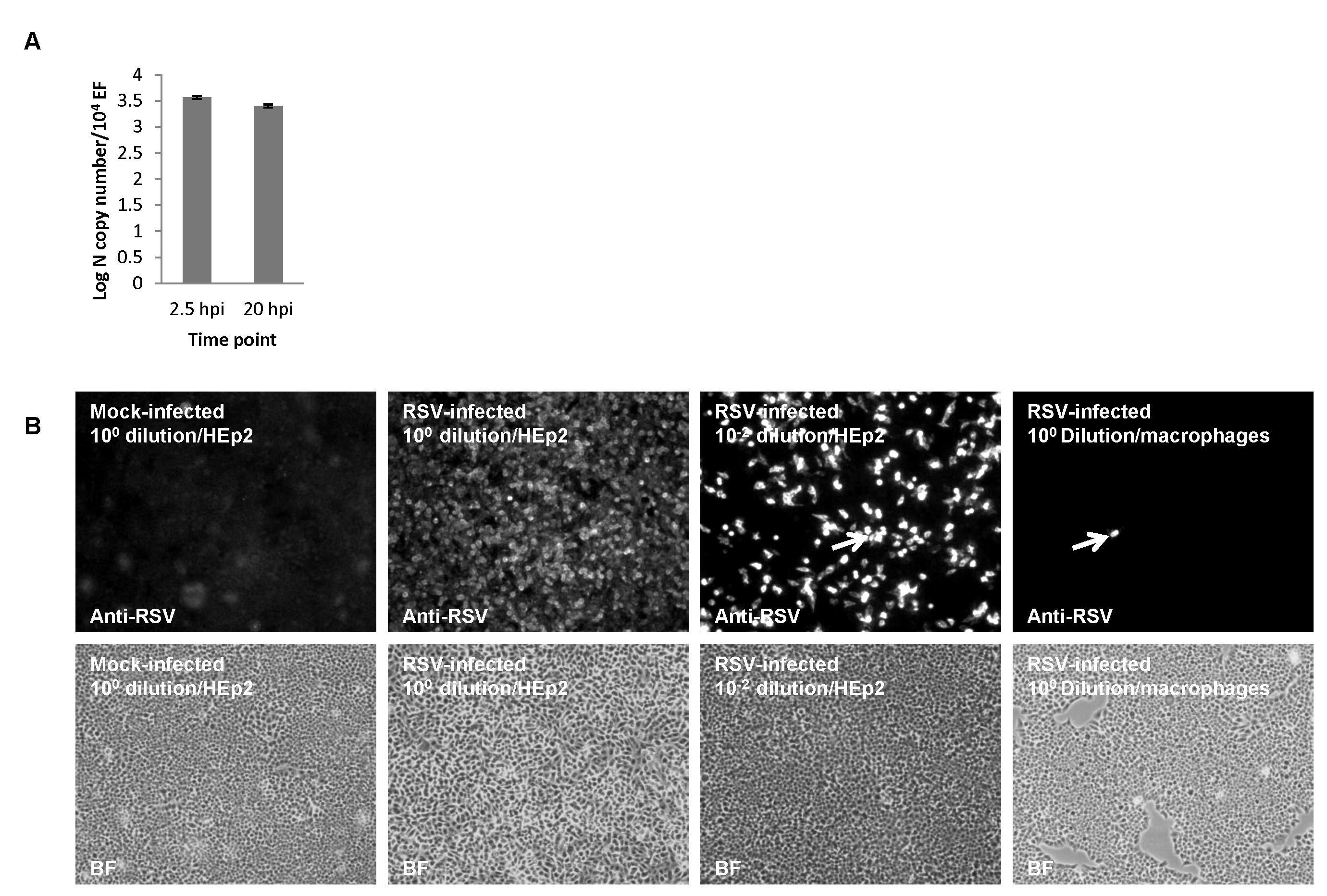

Supplement: Additional file 3: Figure S3 — Infectious virus particles are not produced in RSV-infected macrophages (A) The total RNA was extracted from RSV-infected MΦ cells at 2.5 and 24 hpi and the vRNA levels estimated by qPCR as described in methods. This is the average of 3 measurements and p < 0.05. (B) The tissue culture supernatant (TCS) from mock-infected or RSV-infected HEp2 cells or PMΦ cells was harvested at 24 hpi and used to infect HEp2 monolayers. At 24 hpi the presence of infected cells in the HEp2 cell monolyer was stained using anti-RSV and viewed by fluorescence microscopy (anti-RSV) and bright field microscopy (BF) (objective x10). [file 1471-2164-14-190-S3.tiff]

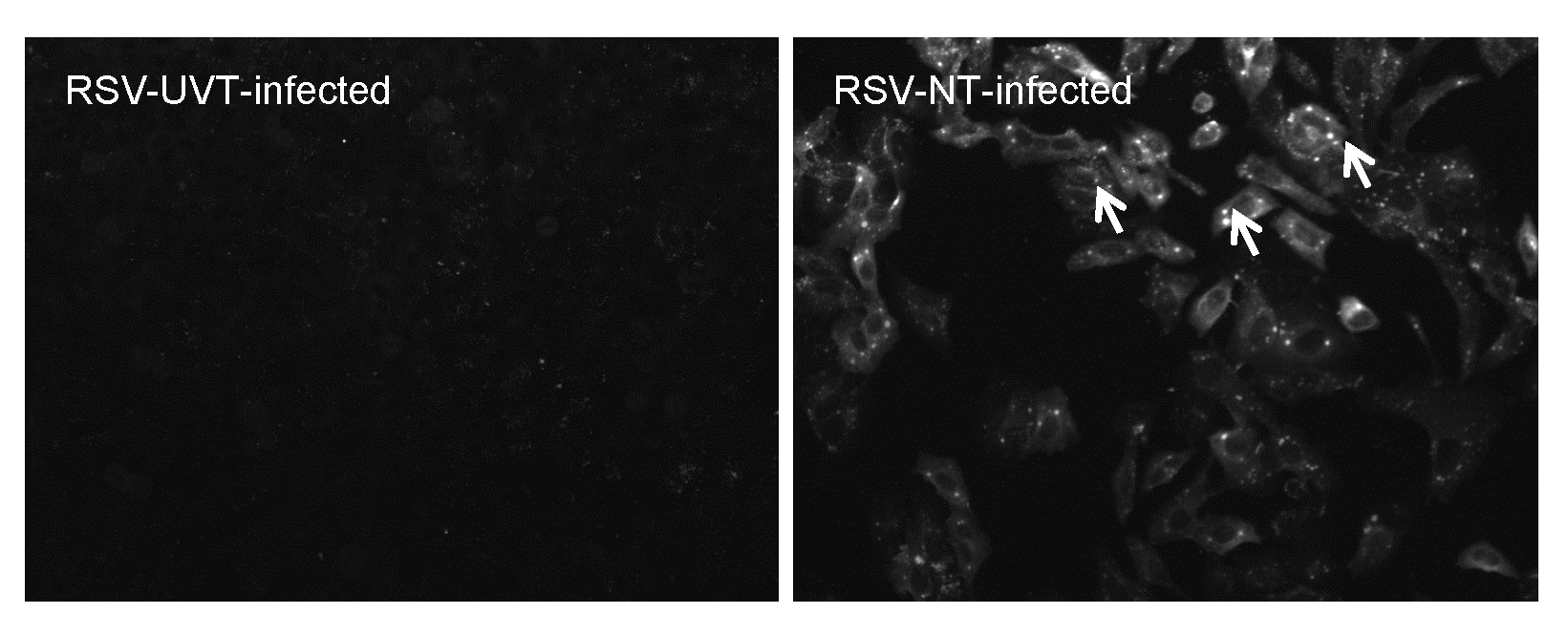

Supplement: Additional file 6: Figure S4 — UV-treatment inactivates RSV infectivity. The RSV inoculum was either non-treated (RSV-NT) or UV-treated (RSV-UVT) and used to infect either HEp2 cells and at 24 hours post-infection the cells were stained using anti-RSV and viewed using a Nikon eclipse 80i fluorescence microscope (objective x10). [file 1471-2164-14-190-S6.tiff]

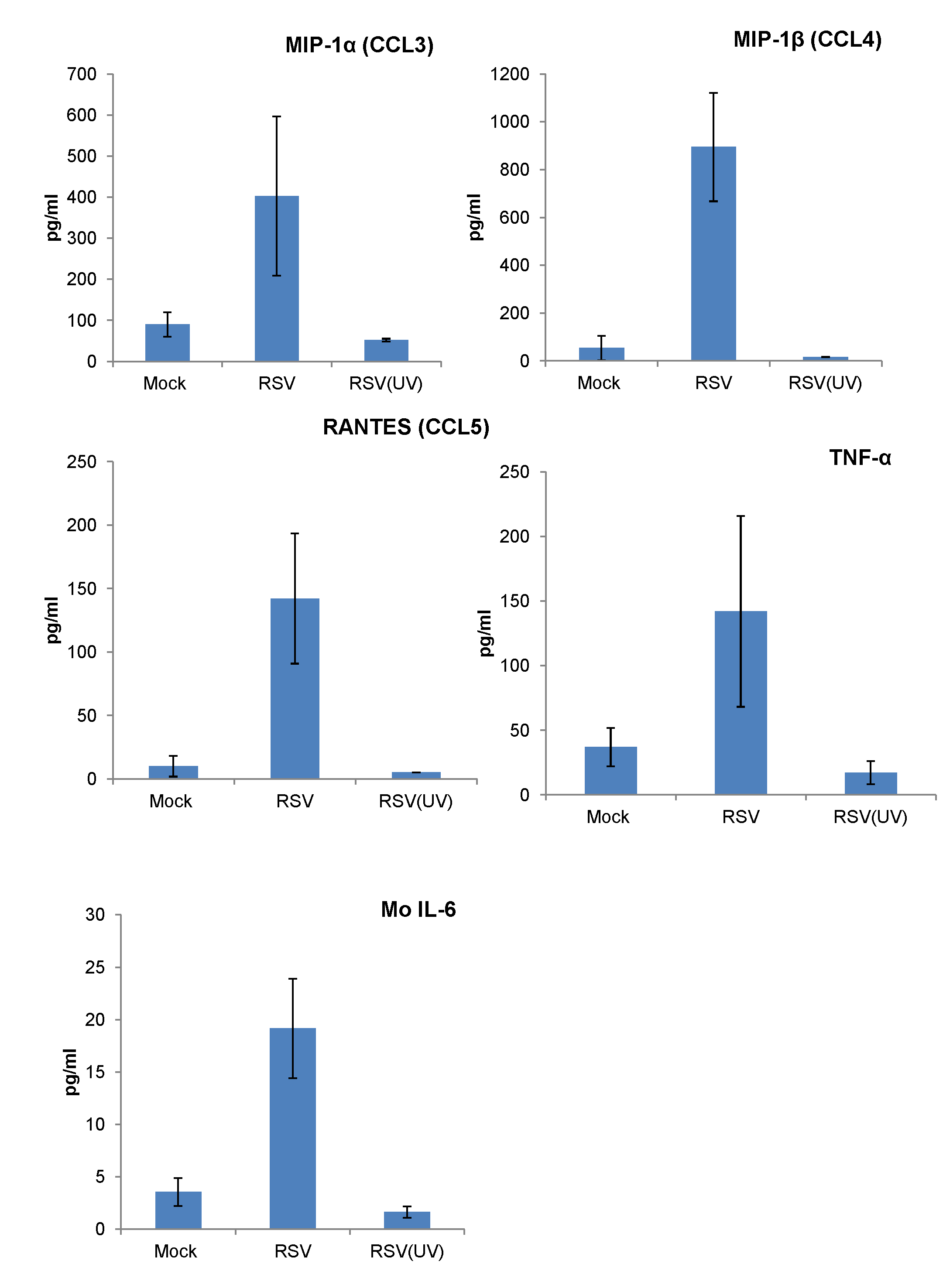

Supplement: Additional file 7: Figure S5 — Cytokine induction in the murine alveolar macrophage (AMФ) cell preparation. The AMФ cell preparation was either mock-infected (Mock) or infected using a RSV inoculum that was either non-treated (RSV) or UV-inactivated (RSV(UV)). The levels of pro-inflammatory cytokines in the tissue culture supernatant (TCS) was measured at 24 hrs post-infection (hpi). In each case the data shown were obtained from triplicate measurements (p < 0.05) and representative data from two independent experiments is shown. [file 1471-2164-14-190-S7.tiff]

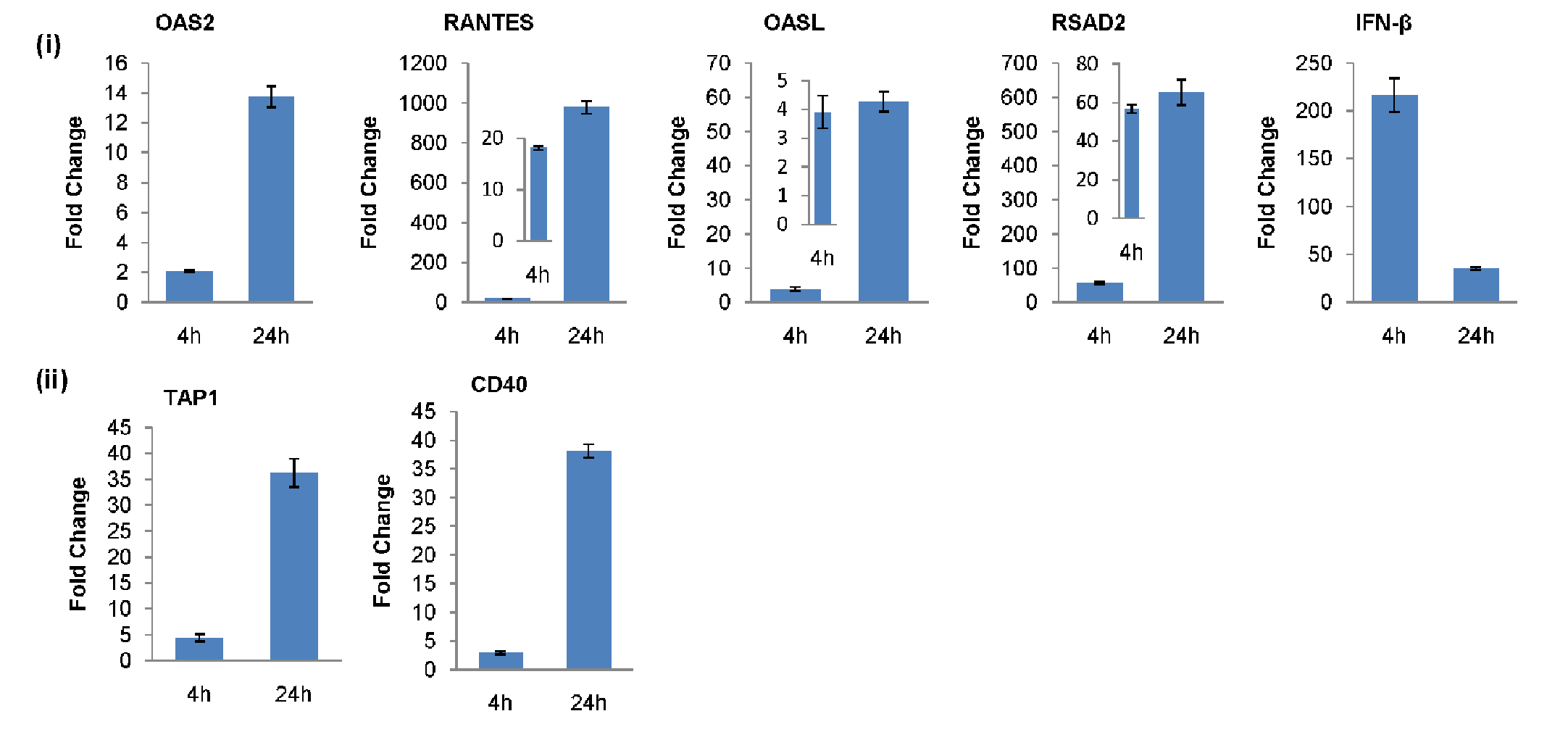

Supplement: Additional file 8: Figure S6 — Validation in the differential gene expression of selected genes in RSV-infected MΦ cells using qPCR. The relative mRNA levels in mock and RSV-infected MΦ cells of (i) 2’,5’-oligoadenylate synthase 2 (OAS2), radical S-adenosyl methionine domain containing 2 (RSAD2), RANTES, Interferon-β (IFNβ) and 2’,5’-oligoadenylate synthase-like genes (OASL) (insets show expression levels in mock-infected cells) and (ii) TAP1, CD40. The average values obtained from three independent measurements (p < 0.05) and representative data from one experiment. [file 1471-2164-14-190-S8.tiff]
