# Supplementary material for: A systems-based approach to analyse the host response in murine lung macrophages challenged with respiratory syncytial virus
Source: BMC Genomics. 2013 Mar 18;14:190. doi: 10.1186/1471-2164-14-190 (PMC3618260; doi:10.1186/1471-2164-14-190)
Supplement: Additional file 9: Table S3 — Primer and probes sequences designed for real-time qPCR. Primer sequences and UPL probes (Roche) used for real-time qPCR validation . RSV N gene, IFN-β1: interferon β; OAS2: 2’, 5’-oligoadenylate synthase 2; OASL: 2’, 5’-oligoadenylate synthase-like; RSAD2: radical S-adenosyl methionine domain containing 2; HMGCR: 3-hydroxy-3-methyl-glutaryl-CoA reductase; CH25H: cholesterol 25-hydroxylase; TAP1: Antigen peptide transporter 1; CD40:CD40; RANTES (CCL5); EF: elongation factor. [file 1471-2164-14-190-S9.doc]

| Origin | Gene | Sequence (5’-3’) | Probe Sequence  (UPL #) |
| --- | --- | --- | --- |
| RSV | N FW | CAGCACTTGTAATAACCAAATTAGCAT | CAGCAGGA (#94) |
|  | N RV | TGCAAAAACATCTTCAAGTCTCTG |  |
| Mouse | OAS2 FW | GGCCCAGGACTTGAGCTAC | CTCTGCCT (#13) |
|  | OAS2 RV | CCATCGTTGCTAGCTGGTCT |  |
|  | OASL FW | GTGGGGAGACTGCATCCTTA | GGCAGAAG (#29) |
|  | OASL RV | GCAGGGAGATCCAGTTTACCT |  |
|  | IFNΒ FW | CTGGCTTCCATCATGAACAA | TCCTGCTG (#18) |
|  | IFNΒ RV | AGAGGGCTGTGGTGGAGAA |  |
|  | RSAD2 FW | GGTCCAACCTTCTGAATTGC | TGGCTGAG (#102) |
|  | RSAD2 RV | ATCAGGGAAAGAGACATGCAG |  |
|  | EF FW | ACACGTAGATTCCGGCAAGT | TGGTGGAA (#31) |
|  | EF RV | ACTTTTCGATGGTTCGCTTG |  |
|  | CD40 FW | CCCTGCTGGTCATTCCTG | TGGGCATC (#105) |
|  | CD40 RV | AGAGAAACACCCCGAAAATG |  |
|  | TAP1 FW | ACCATGGAGGAAATCACAGC | TCTGGAGC (#50) |
|  | TAP1 RV | GGAACCCAGAGATGAAATCG |  |
|  | RANTES FW | CCTACTCCCACTCGGTCCT | TGGGCATC (#105) |
|  | RANTES RV | TTTCTTGGGTTTGCTGTGC |  |

Primer sequence in qPCR measurements.
